# Exploring Co-occurrence patterns and microbial diversity in the lung microbiome of patients with non-small cell lung cancer

**DOI:** 10.1186/s12866-023-02931-9

**Published:** 2023-07-11

**Authors:** Sadaf Najafi, Sadegh Azimzadeh Jamalkandi, Ali Najafi, Jafar Salimian, Ali Ahmadi

**Affiliations:** 1 Department of Biostatistics, Faculty of Medical Sciences, Tarbiat Modares University, Tehran, Iran; 2 Chemical Injuries Research Center, Systems Biology and Poisonings Institute, Baqiyatallah University of Medical Sciences, Tehran, Iran; 3 Molecular Biology Research Center, Systems Biology and Poisonings Institute, Baqiyatallah University of Medical Sciences, Tehran, Iran; 4 Applied Virology Research Center, Systems Biology and Poisonings Institute, Baqiyatallah University of Medical Sciences, Tehran, Iran

**Keywords:** Lung cancer, Lung microbiome, 16S rRNA gene sequencing, Microbiome diversity, Co-occurrence network, Microbiome data integration

## Abstract

**Background:**

It has been demonstrated in the literature that a dysbiotic microbiome could have a negative impact on the host immune system and promote disease onset or exacerbation. Co-occurrence networks have been widely adopted to identify biomarkers and keystone taxa in the pathogenesis of microbiome-related diseases. Despite the promising results that network-driven approaches have led to in various human diseases, there is a dearth of research pertaining to key taxa that contribute to the pathogenesis of lung cancer. Therefore, our primary goal in this study is to explore co-existing relationships among members of the lung microbial community and any potential gained or lost interactions in lung cancer.

**Results:**

Using integrative and network-based approaches, we integrated four studies assessing the microbiome of lung biopsies of cancer patients. Differential abundance analyses showed that several bacterial taxa are different between tumor and tumor-adjacent normal tissues (FDR adjusted p-value < 0.05). Four, fifteen, and twelve significantly different associations were found at phylum, family, and genus levels. Diversity analyses suggested reduced alpha diversity in the tumor microbiome. However, beta diversity analysis did not show any discernible pattern between groups. In addition, four distinct modules of bacterial families were detected by the DBSCAN clustering method. Finally, in the co-occurrence network context, *Actinobacteria*, *Firmicutes*, *Bacteroidetes*, and *Chloroflexi* at the phylum level and *Bifidobacterium*, *Massilia*, *Sphingobacterium*, and *Ochrobactrum* at the genus level showed the highest degree of rewiring.

**Conclusions:**

Despite the absence of statistically significant differences in the relative abundance of certain taxa between groups, it is imperative not to overlook them for further exploration. This is because they may hold pivotal central roles in the broader network of bacterial taxa (e.g., *Bifidobacterium* and *Massilia*). These findings emphasize the importance of a network analysis approach for studying the lung microbiome since it could facilitate identifying key microbial taxa in lung cancer pathogenesis. Relying exclusively on differentially abundant taxa may not be enough to fully grasp the complex interplay between lung cancer and the microbiome. Therefore, a network-based approach can offer deeper insights and a more comprehensive understanding of the underlying mechanisms.

**Supplementary Information:**

The online version contains supplementary material available at 10.1186/s12866-023-02931-9.

## Background

Lung cancer is a global challenge causing over 1.3 million deaths per year. The histology of lung cancer is heterogeneous, with different patterns of progression and prognosis, and is usually diagnosed in advanced stages, with a survival rate of about five years after diagnosis [1]. Therefore, identifying early-stage patients with a higher risk of disease recurrence can increase the survival rate of lung cancer [2]. As demonstrated, the microbiome populations residing in lower airways may get imbalanced in the structure and composition (dysbiosis state) during respiratory diseases such as Asthma, COPD, and lung cancer [3]. Accordingly, metagenomic studies have demonstrated an association between lung microbiome dysbiosis and lung cancer [4, 5]. Cameron et al. suggested potential bacterial biomarkers for lung cancer in sputum microbiome samples and showed that *Streptococcus viridans* and *Granulicatella adiacens* were significantly higher in lung cancer [1]. Yu et al. reported higher levels of *Thermus* and lower levels of *Ralstonia* in tumor tissues from patients with advanced lung cancer compared with non-malignant lung tissues, suggesting an essential role for these bacteria in lung cancer progression [4]. In addition, Lee et al. detected an increased abundance of *Veillonella* and *Megasphaera* in bronchoalveolar lavage fluid specimens of lung cancer patients [6]. These changes in the microbiome community may reflect biochemical changes in the lungs of cancer patients, which are associated with an increased anaerobic environment and altered metabolism of pyridoxal/polyamine and nitrogen.

Most of the prior studies have mainly focused on the differentially abundant taxa, and whether these taxa play a key role in lung cancer development or progression remains to be answered. To fully understand the contributions of these taxa to lung cancer, it is crucial to understand the structure of the lung microbial community through co-occurrence networks [7], in which nodes represent bacterial taxa and edges represent the interdependent relationships amongst them [8]. Accordingly, new studies have used a network analysis approach to visually represent the interrelations among lung microbial community members. This approach can help to identify key taxa and shed light on the contribution of specific taxa to the functioning of a particular ecosystem. They try to find the behavior of the microbiome as an integrated network rather than in an individual manner in various diseases. For example, Greenblum et al. conducted a study that integrated gut microbiome metagenomic data for obesity and inflammatory bowel disease (IBD) to create community-level metabolic networks. Their findings showed that differences in gene-level and network-level topology associated with these two diseases tend to occur at the periphery of the metabolic network and are enriched for topologically derived metabolic inputs. They also indicated that the microbiome of obese individuals is less modular [9]. Layeghifard et al. introduced an unsupervised approach to identify key taxa in the sputum microbiome of cystic fibrosis patients. They defined key taxa based on relative abundance, prevalence, and co-occurrence network interconnectedness. They found that taxa with the highest network interconnectedness tracked changes in patient health significantly better than taxa with the highest abundance or prevalence. In addition, they found that network interconnectedness most strongly delineated the taxa among clinical states and that anaerobic bacteria were over-represented during cystic fibrosis exacerbations [10]. Einarsson et al. also performed a co-occurrence network analysis of the microbiome of the human airways. They revealed a core community structure with several key microbiome taxa critical to health and disease [11].

Conflicting results from existing studies in microbiome research can often be attributed to factors such as the personalized nature of the microbiome, small sample sizes, and variations across studies platforms. Researchers should consider combining multiple datasets to overcome these challenges and achieve more robust and reliable outcomes. In the previous study, we performed a meta-analysis on several lung microbiome datasets to integrate and evaluate the alterations of the lung microbiome in lung cancer patients [12]. In this study, we applied an integrative and systems biology approach to provide a more comprehensive understanding of the interaction between the lung microbiome and lung cancer. By integrating the same sets of 16S rRNA sequencing data on lung biopsies from our previous study, our analysis aimed to compare the diversity indices and clustering properties of the lung microbiome between cancer and non-cancer biopsy samples and reconstruct the co-occurrence network for the lung microbial community in lung cancer.

## Results

### Datasets characteristics

**Fig. 1:**
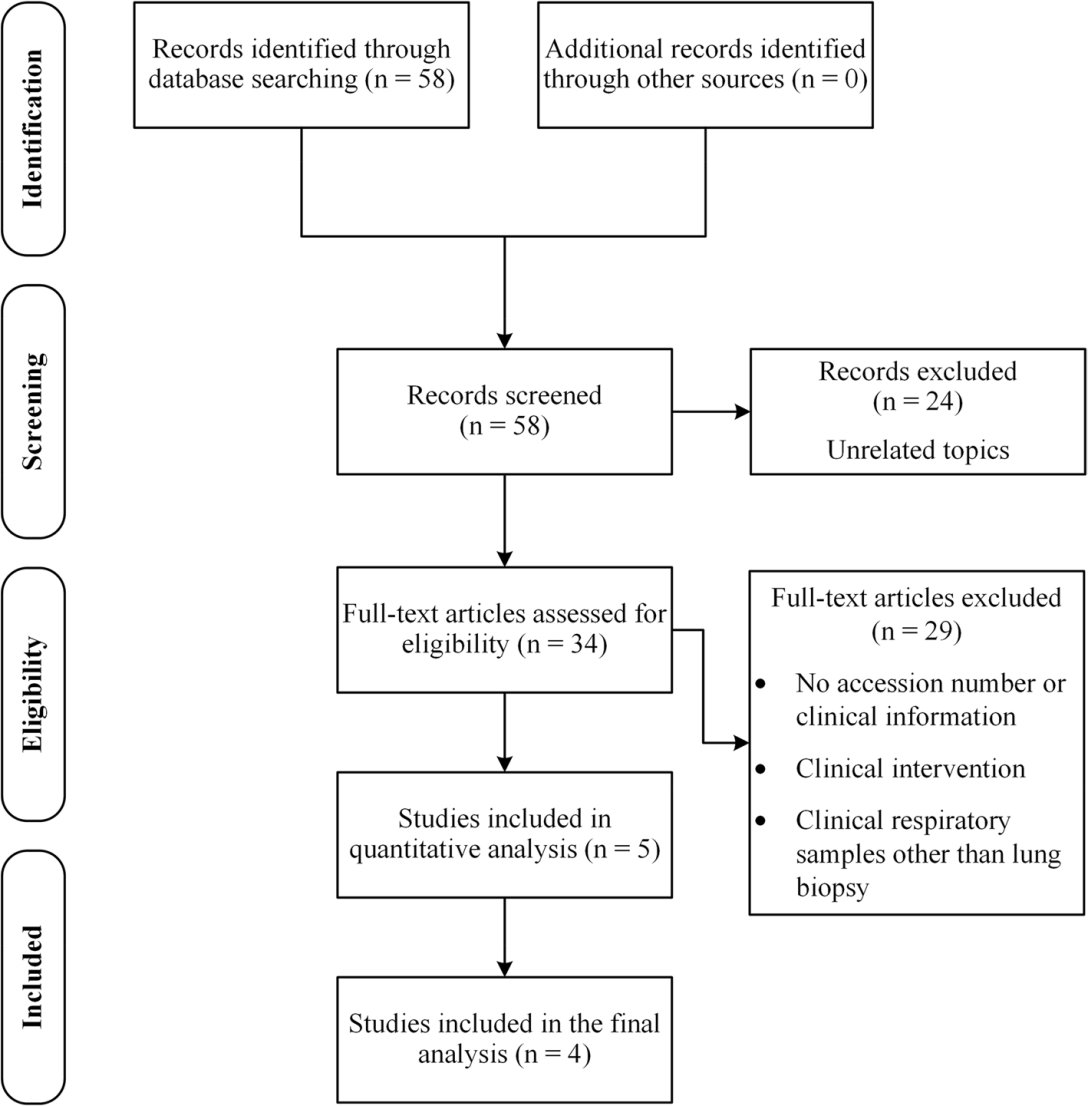
Systematic search process

The systematic search and study selection process, along with exclusion criteria, were summarized in Fig. 1. The main characteristics of the included studies are presented in Table 1. In all the included studies, lung biopsy specimens have been used, which were taken during surgical or bronchoscopy procedures. Lung tumor tissues were matched with tumor-adjacent normal tissues (located as far as possible with no evidence of tumor nuclei) of the same patient. As can be seen from the table, we restricted our study to integrating studies with a similar design and experimental procedure. The included studies were homogeneous in terms of the amplified hypervariable region of the 16S rRNA gene, sequencing platform, and age. The raw data of the studies mentioned above were then processed using a standard pipeline to minimize inter-study batch effects. The case group included tumor tissues related to two subtypes of NSCLC, including adenocarcinoma and squamous cell carcinoma; tumor-adjacent normal tissues were considered the control group.

**Table 1:** Characteristics of included studies

Datasets	Accession	16S HV region	Sequencing platform	Case (No.)	Control (No.)	Age (YO.)	References
1	PRJNA624822	*	*	245	231	40–90	[13]
2	PRJNA303190	V3–V4	Illumina MiSeq	50	207	40–80	[4]
3	PRJNA327258	NA	Illumina MiSeq	27	26	40–80	**
4	PRJNA647170	V3–V4	Illumina MiSeq	25	24	52–72	[14]
				**347**	**488**		

Case: lung tumor tissues, Control: tumor-adjacent normal tissues, NA: Not Available, * Five regions of the 16S rRNA gene were amplified and sequenced on Illumina HiSeq, MiSeq, or NextSeq. ** Dataset 2 (published) and Dataset 3 (unpublished) were separately available in SRA. With further assessment, we noticed both datasets had been taken from the same population. Therefore, we omitted the repeated samples and considered these two separate studies

### Taxonomic diversity in lung cancer

**Fig. 2:**
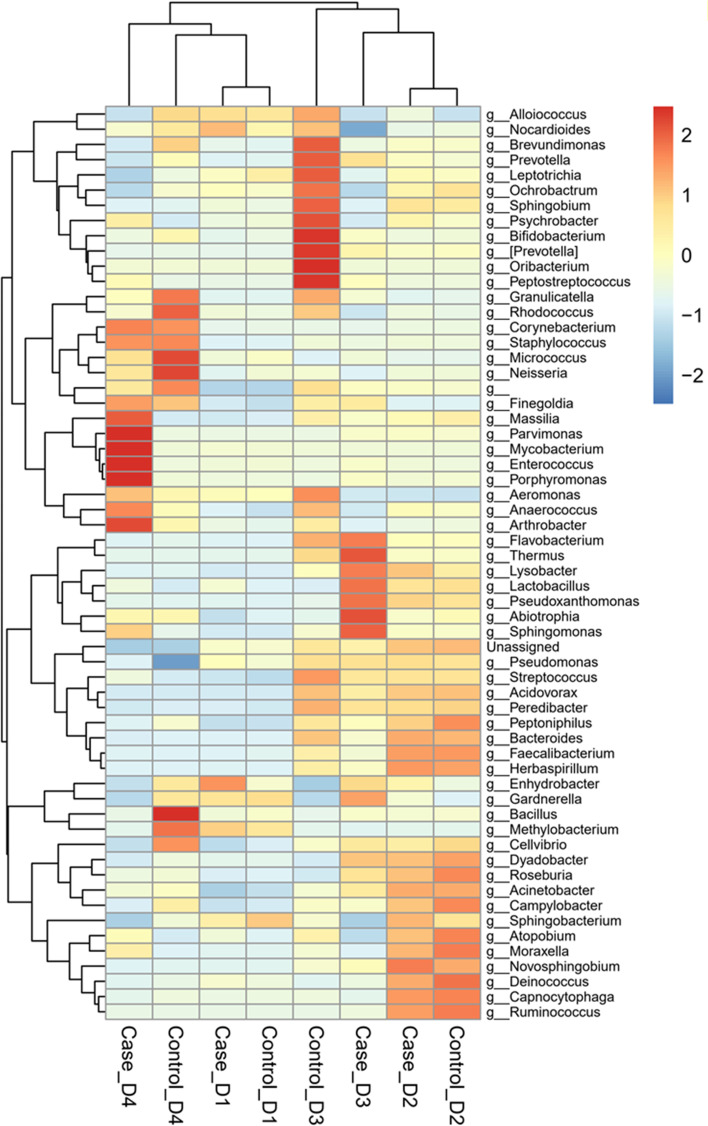
Scaled relative frequency of shared genera across different datasets. The heatmap displays the variation in the microbiome composition at the genus level. The average relative frequency of the shared genera has been shown. D1: Dataset 1, D2: Dataset 2, D3: Dataset 3, D4: Dataset 4

To determine the common microbiome composition of four selected datasets, the relative frequency tables of all datasets were merged and analyzed based on shared microbial taxa at the phylum, family, and genus levels. The results for the genus level have been presented in Fig. 2. Further results for phylum and family levels are shown in Additional file 1.

**Fig. 3:**
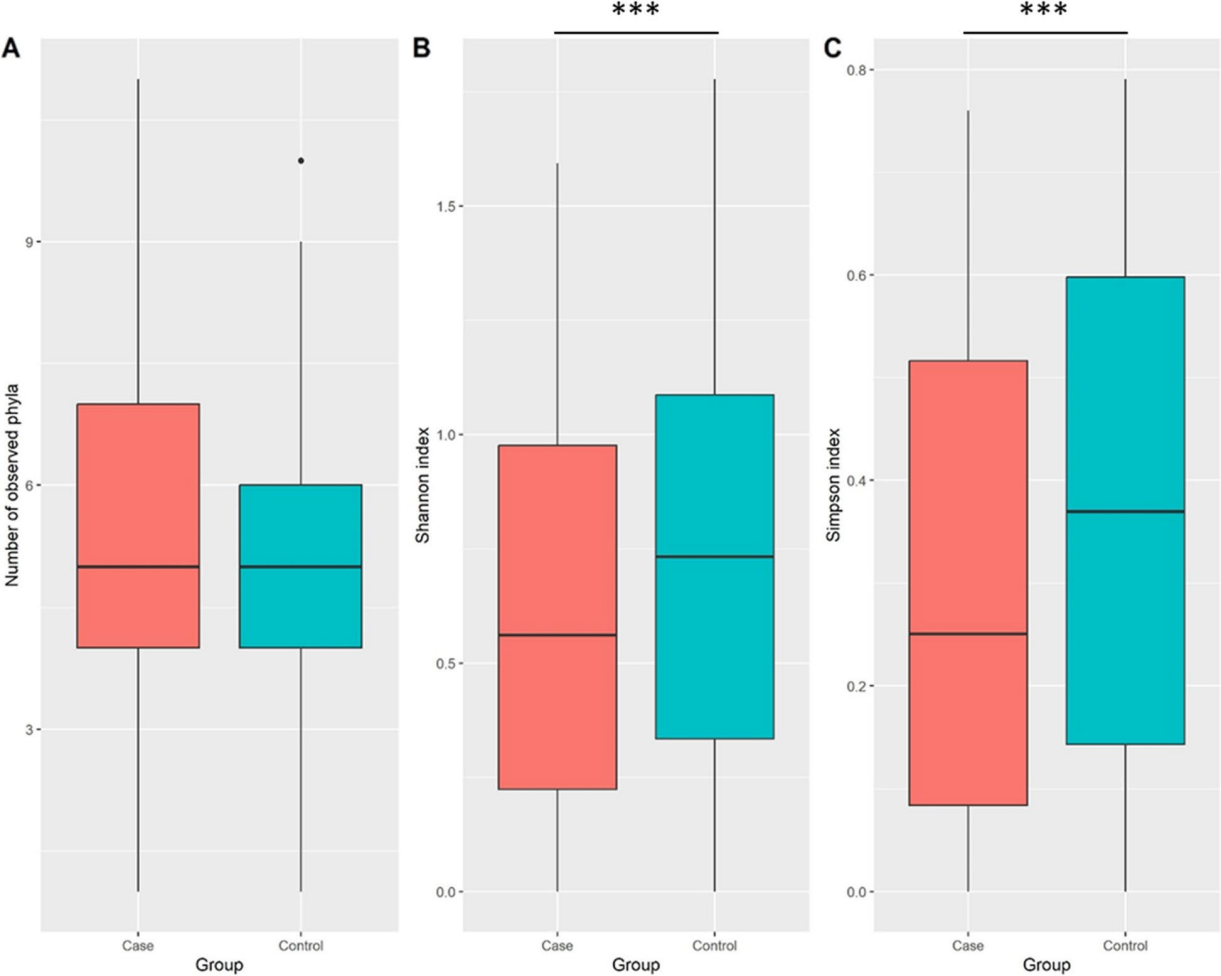
Alpha diversity at the phylum level. **A** Observed phyla **B** Shannon diversity index (aka Shannon–Wiener index) **C** Simpson index. Boxplots summarize estimated alpha diversity based on different metrics within each group and show differences between cases and controls. Asterisks represent a significant result from the Wilcoxon Rank Sum Test. Case: lung tumor tissues, Control: tumor-adjacent normal tissues, ***p < 0.001

**Fig. 4:**
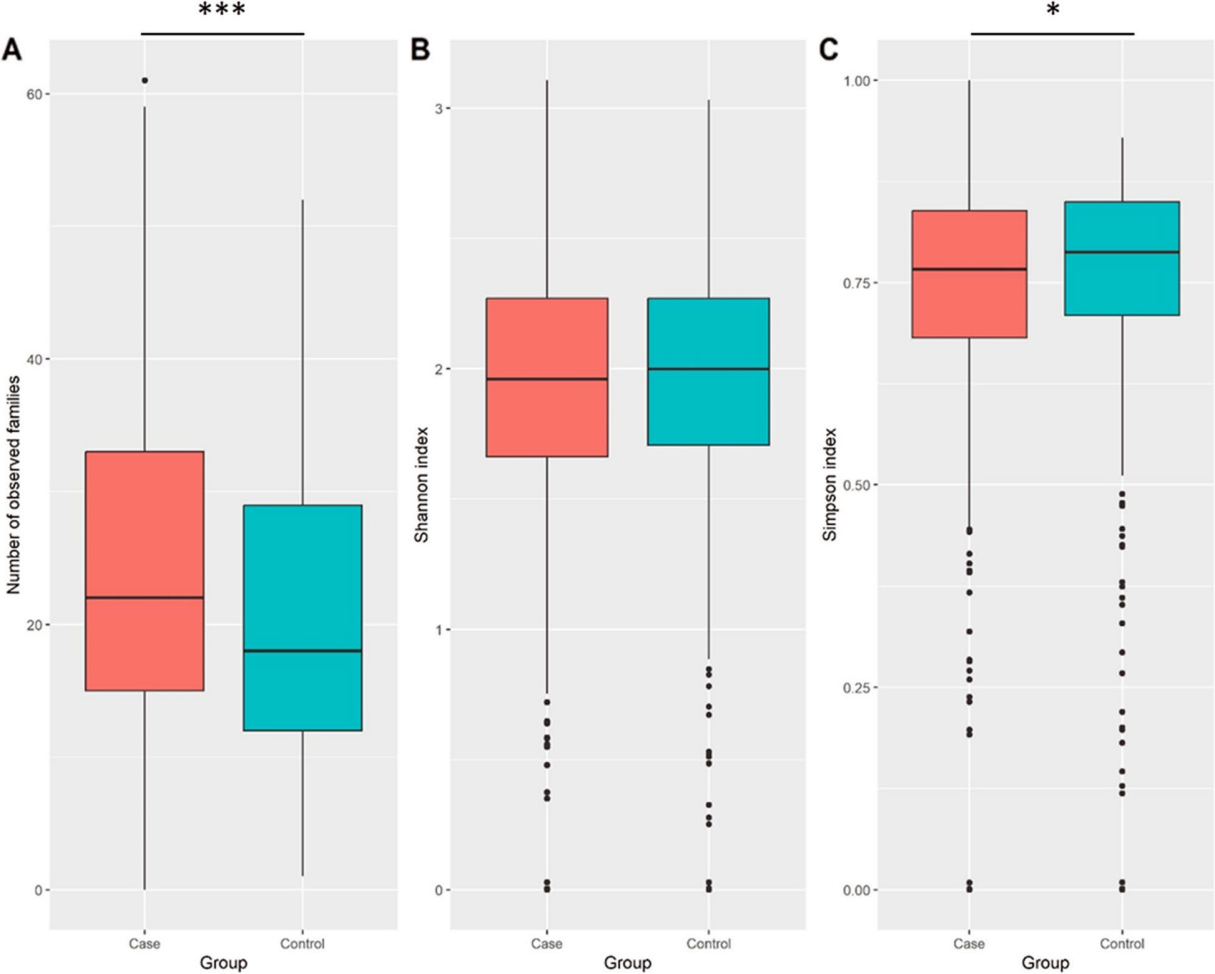
Alpha diversity at the family level. **A** Observed families **B** Shannon diversity index (aka Shannon–Wiener index) **C** Simpson index. Boxplots summarize estimated alpha diversity based on different metrics within each group and show differences between cases and controls. Asterisks represent a significant result from the Wilcoxon Rank Sum Test. Case: lung tumor tissues, Control: tumor-adjacent normal tissues, *p < 0.05, ***p < 0.001

**Fig. 5:**
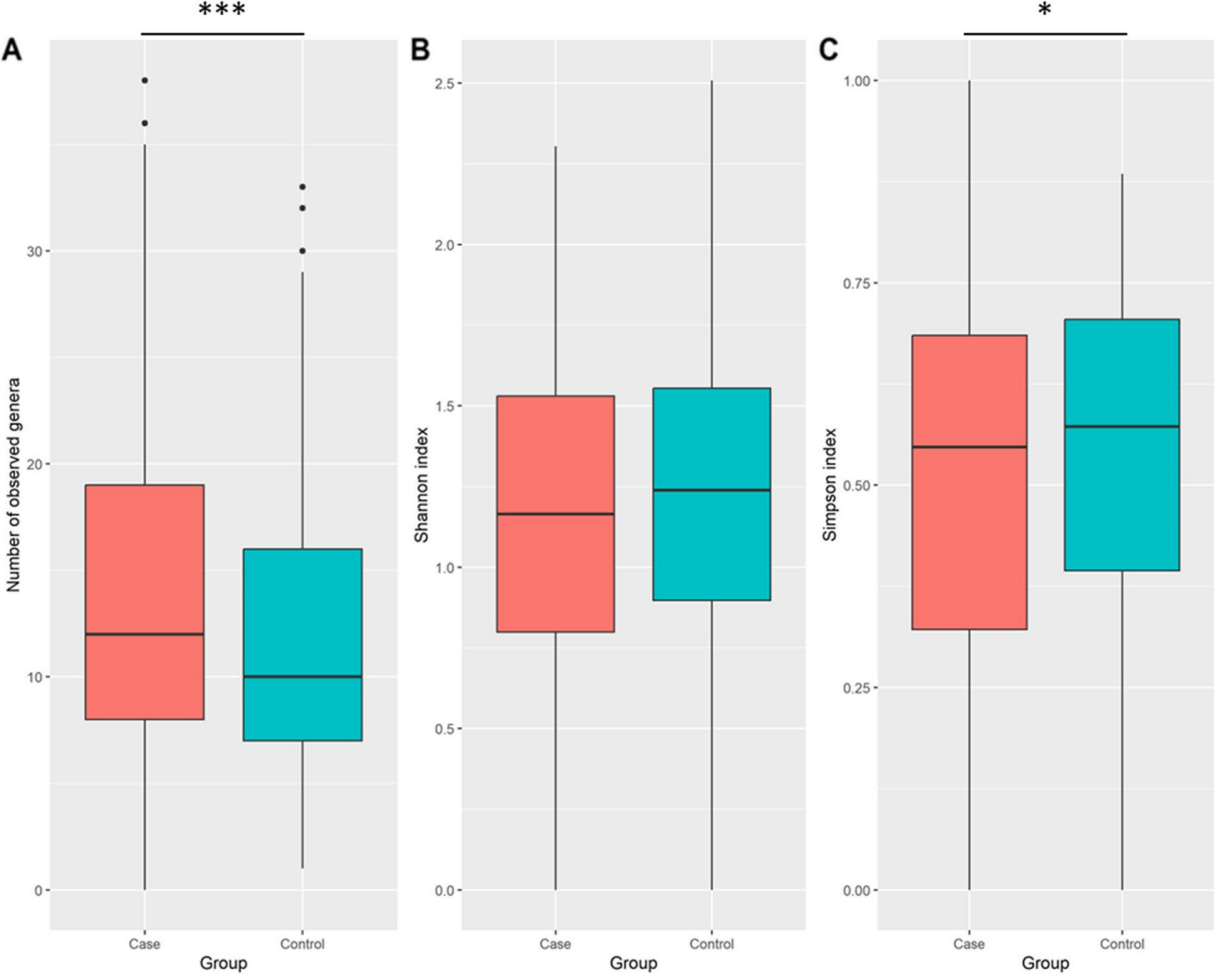
Alpha diversity at the genus level. **A** Observed genera **B** Shannon diversity index (aka Shannon–Wiener index) **C** Simpson index. Boxplots summarize estimated alpha diversity based on different metrics within each group and show differences between cases and controls. Asterisks represent a significant result from the Wilcoxon Rank Sum Test. Case: lung tumor tissues, Control: tumor-adjacent normal tissues, *p < 0.05, ***p < 0.001

**Fig. 6:**
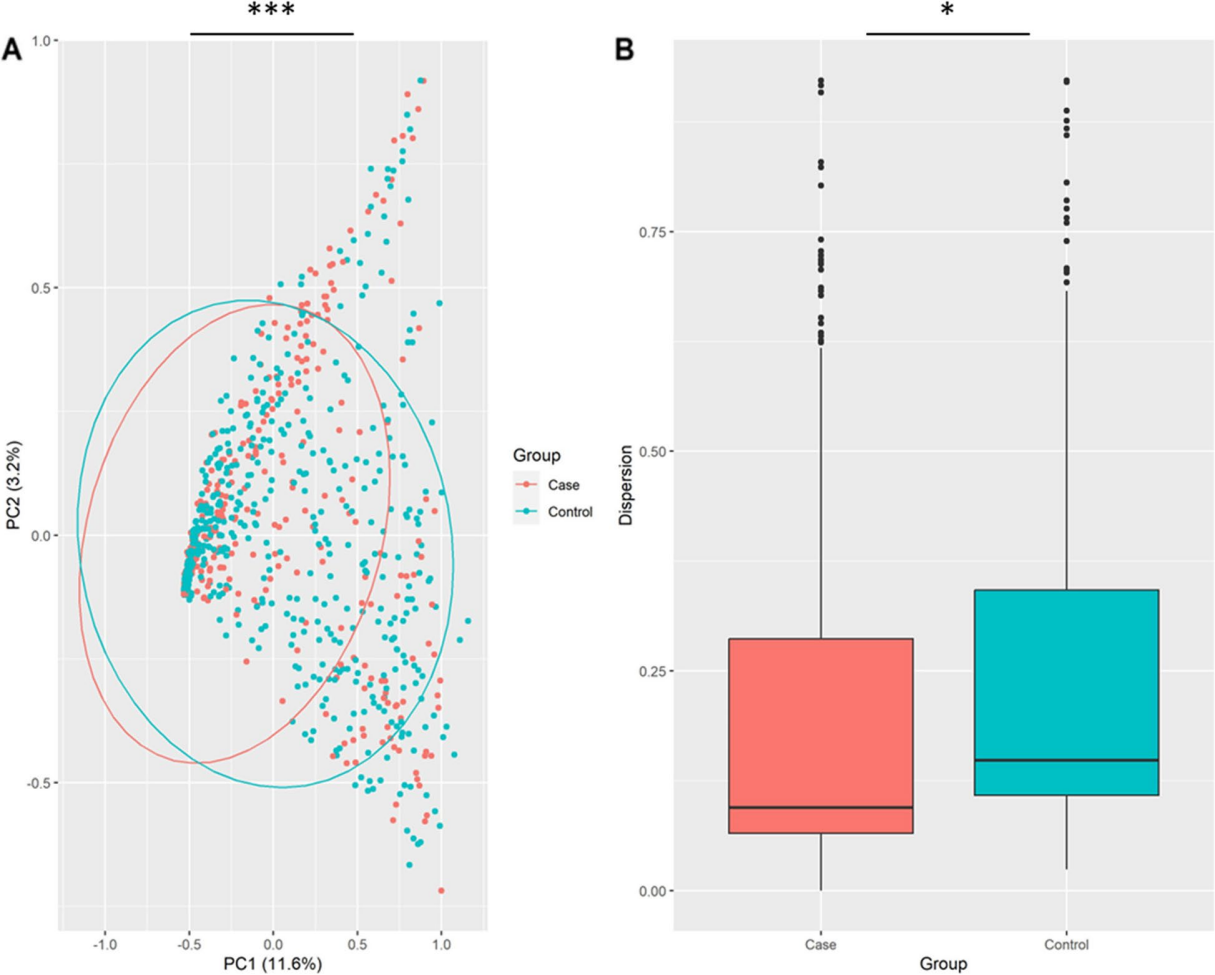
Beta diversity at the phylum level. **A** PCoA plot based on Bray–Curtis dissimilarity shows the differences in the lung microbiome composition between cases and controls. Each dot represents the microbiome of a sample, and asterisks indicate a significant result from PERMANOVA. **B** Boxplots show dispersion within and between groups. Asterisks denote a significant result from ANOVA. Case: lung tumor tissues, Control: tumor-adjacent normal tissues, *p < 0.05, ***p < 0.001

**Fig. 7:**
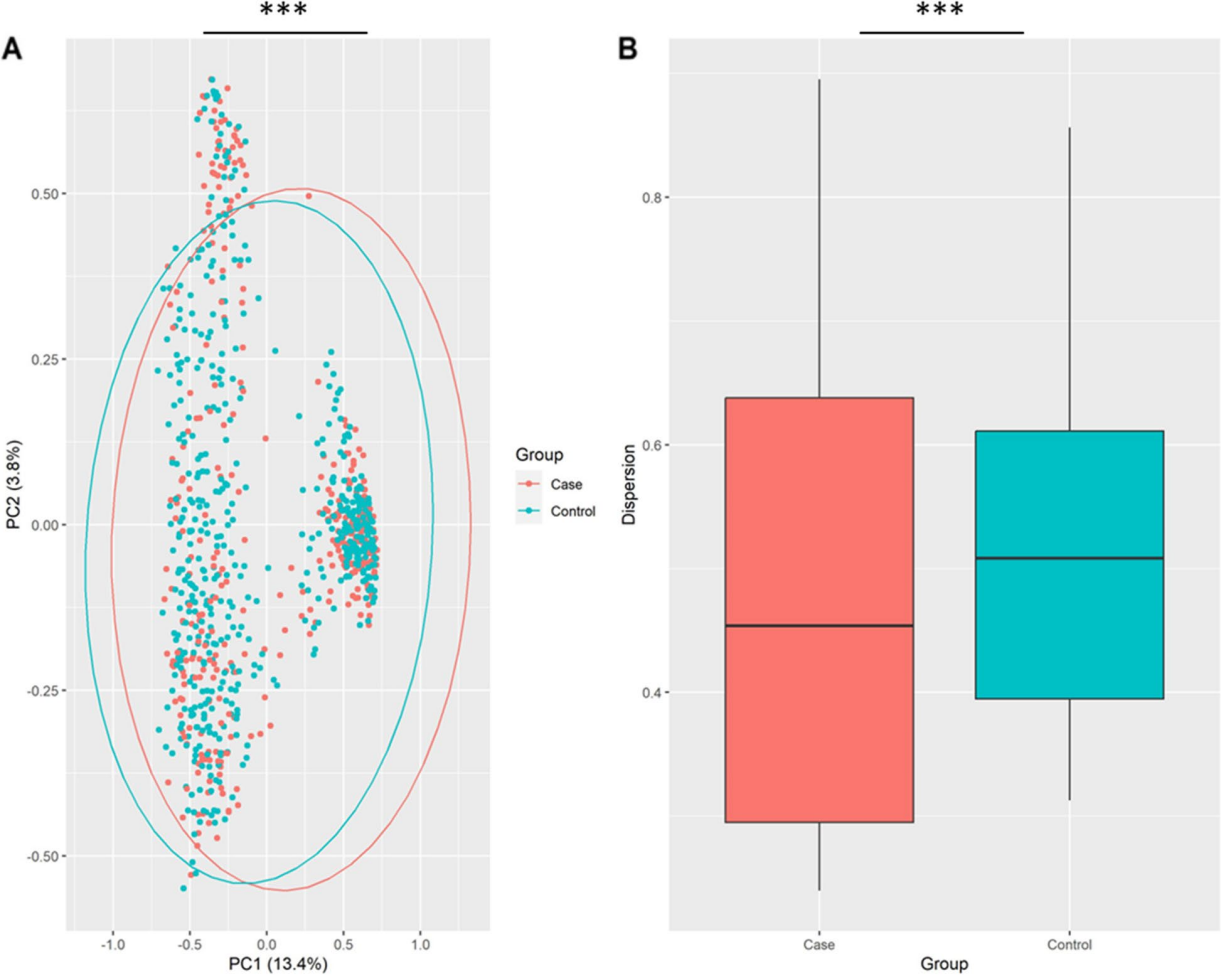
Beta diversity at the family level. **A** PCoA plot based on Bray–Curtis dissimilarity shows the differences in the lung microbiome composition between cases and controls. Each dot represents the microbiome of a sample, and asterisks indicate a significant result from PERMANOVA. **B** Boxplots show dispersion within and between groups. Asterisks denote a significant result from ANOVA. Case: lung tumor tissues, Control: tumor-adjacent normal tissues, ***p < 0.001

**Fig. 8:**
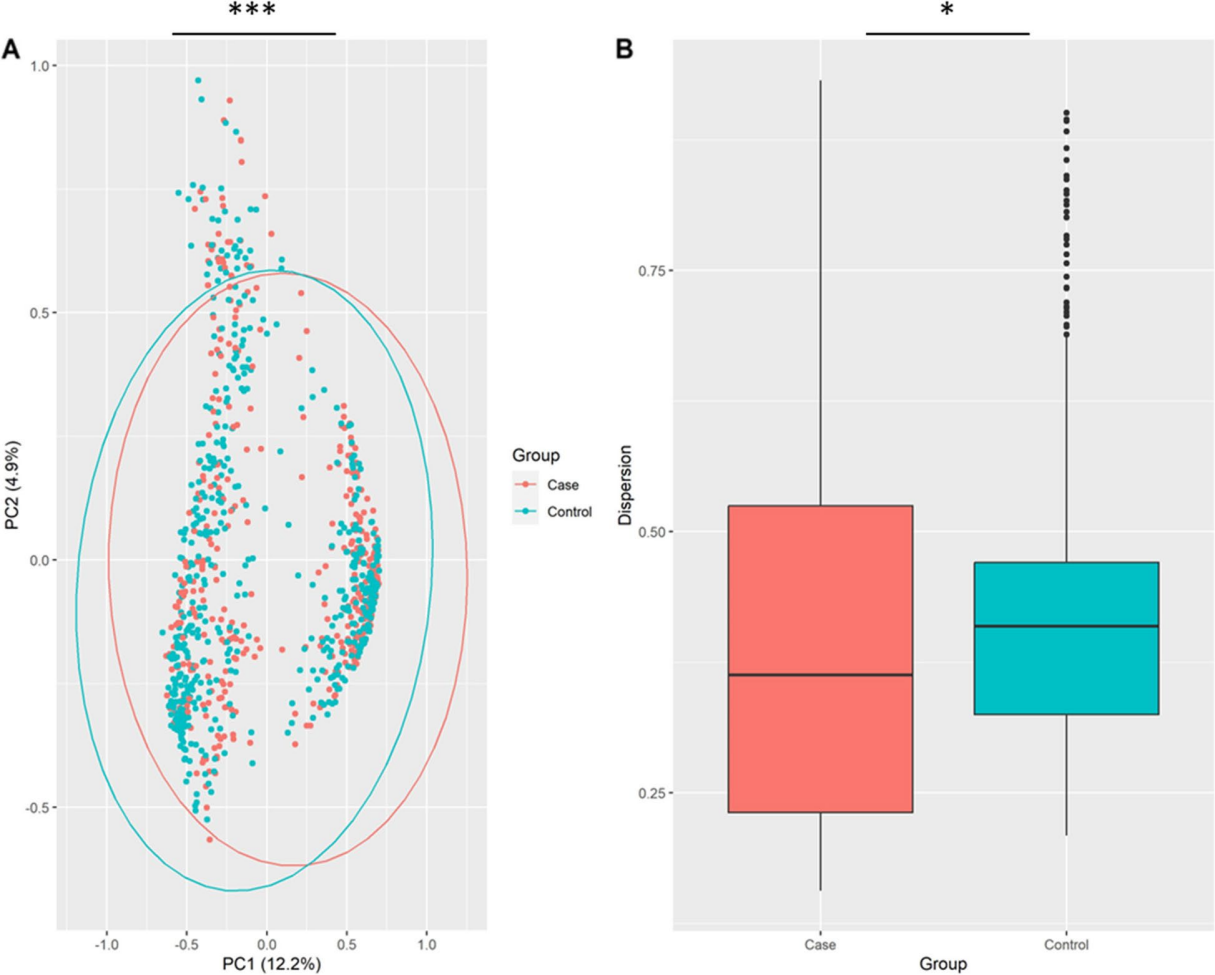
Beta diversity at the genus level. **A** PCoA plot based on Bray–Curtis dissimilarity shows the differences in the lung microbiome composition between cases and controls. Each dot represents the microbiome of a sample, and asterisks indicate a significant result from PERMANOVA. **B** Boxplots show dispersion within and between groups. Asterisks denote a significant result from ANOVA. Case: lung tumor tissues, Control: tumor-adjacent normal tissues, *p < 0.05, ***p < 0.001

In the first phase of diversity analyses, we examined alpha diversity, which focuses on diversity within a sample. The calculated Shannon and Simpson indices were visualized using Boxplots. Figures 3, 4 and 5 provide the resulting Boxplots at various taxonomic levels. A Wilcoxon Rank Sum Test was used to further investigate if sample type (tumor vs. tumor-adjacent normal tissue) is associated with the variation of diversity indices. As shown in Fig. 3, a statistically significant difference between the two groups was more evident at the phylum level. Family and genus levels also displayed some differences in alpha diversity between groups. These results suggest a reduced diversity in tumor tissues compared to normal tissues. In the second phase and to investigate how the overall taxonomic composition of the lung microbiome differs between groups, we estimated beta diversity, which is quantified based on dissimilarities among samples. We employed the Bray–Curtis index as a commonly used beta diversity measure for all pairs of samples based on the relative abundance values. Since we are dealing with high-dimensional data, we need to retain a limited number of dimensions (usually the first two or three) for data visualization. Principal Coordinates Analysis was adopted for this purpose, and according to its results, Figs. 6, 7 and 8, no distinct pattern was observed across different taxonomic levels between case and control groups, suggesting the disease may not dramatically alter the structure of the lung microbial community. To test whether the group variable could explain the variation in microbiome composition between the two communities, we performed a PERMANOVA test, and the results suggested that the microbiome composition is significantly different between groups. However, closer inspection of the results shows that the two groups have significantly different multivariate dispersions. In other words, the assumption of variance homogeneity between groups does not hold, partly explaining the PERMANOVA test results.

Additionally, the results of diversity for each dataset are as follows. For the alpha diversity analysis: dataset 1 did not show any significant difference between the case and control groups at any taxonomic levels. In dataset 2, only at the genus level, the Simpson index showed a statistically significant difference indicating an increase in alpha diversity in the case group. In dataset 3, only at the family level, the richness of observed families was significantly different between the two groups, suggesting an increase in the richness of the case group. Finally, in dataset 4, the Shannon and Simpson indices were significantly different between the two groups at all three taxonomic levels (phylum, family, and genus), indicating a decrease in the alpha diversity of the case group.

Furthermore, the results of beta diversity analysis did not show any significant differences in the microbial composition between the case and control groups at the phylum level. These differences were only significant in dataset 3 at the family and genus levels. All the results of diversity analyses in individual datasets, along with their corresponding plots, have been presented in Additional file 2.

### Differentially abundant taxa and detected clusters

The statistically significant differences (FDR adjusted *p*-value < 0.05) between cases and controls at phylum, family, and genus levels were summarized in Table 2. The two groups showed the differential abundance of several bacterial phyla (*n* = 4), families (*n* = 15), and genera (*n* = 12). The DBSCAN clustering method was implemented at the family level. This method could detect four distinct modules of bacterial families shown in Table 3. Only 24 nodes were included in the clusters, but the rest of the nodes in the network were not clustered. DBSCAN could not detect any modules at the genus level.

**Table 2:** Significant differences in the relative abundance of bacterial taxa between cases and controls

Phyla (adjusted *p*-value)	Families (adjusted *p*-value)	Genera (adjusted *p*-value)
Bacteroidetes (0.0002)	Weeksellaceae (0.044)	Bacteroides (0.030) Flavobacterium (0.030)
	Bacteroidaceae (0.044)	
	Chitinophagaceae (0.004)	
Proteobacteria (0.001)	Bradyrhizobiaceae (0.0003)	Enhydrobacter (0.004) Methylobacterium (0.006) Sphingomonas (0.006) Brevundimonas (0.030) Herbaspirillum (0.032)
	Caulobacteraceae (0.004)	
	Enterobacteriaceae (0.039)	
	Alcaligenaceae (0.028)	
	Methylobacteriaceae (0.031)	
	Hyphomicrobiaceae (0.005)	
OD1 (0.005)	-	-
Firmicutes (0.017)	Staphylococcaceae (0.004)	Lactobacillus (0.006)
	Lactobacillaceae (0.008)	
The corresponding phyla were not statistically significant	of the phylum Actinobacteria: Mycobacteriaceae (0.006) Nocardioidaceae (0.027) Micrococcaceae (0.039) of the phylum Deinococcota: Deinococcaceae (0.031)	of the phylum Actinobacteria: Mycobacterium (0.006) Nocardioides (0.032) Gardnerella (0.028) of the phylum Deinococcota: Deinococcus (0.030)

**Table 3:** Four modules of bacterial families detected by DBSCAN

Modules	Bacterial families
Module A	Alteromonadaceae, Oxalobacteraceae, Brucellaceae, Aurantimonadaceae
Module B	Veillonellaceae, Coriobacteriaceae, Clostridiaceae, Ruminococcaceae, Porphyromonadaceae, Paenibacillaceae, Flavobacteriaceae, Deinococcaceae, Beijerinckiaceae, Rhodobacteraceae, Lachnospiraceae, Enterococcaceae, Phyllobacteriaceae, Moraxellaceae
Module C	Comamonadaceae, Rhodocyclaceae
Module D	Thermaceae, Verrucomicrobiaceae, Parachlamydiaceae, Legionellaceae

### Rewired Co-occurrence networks

**Fig. 9:**
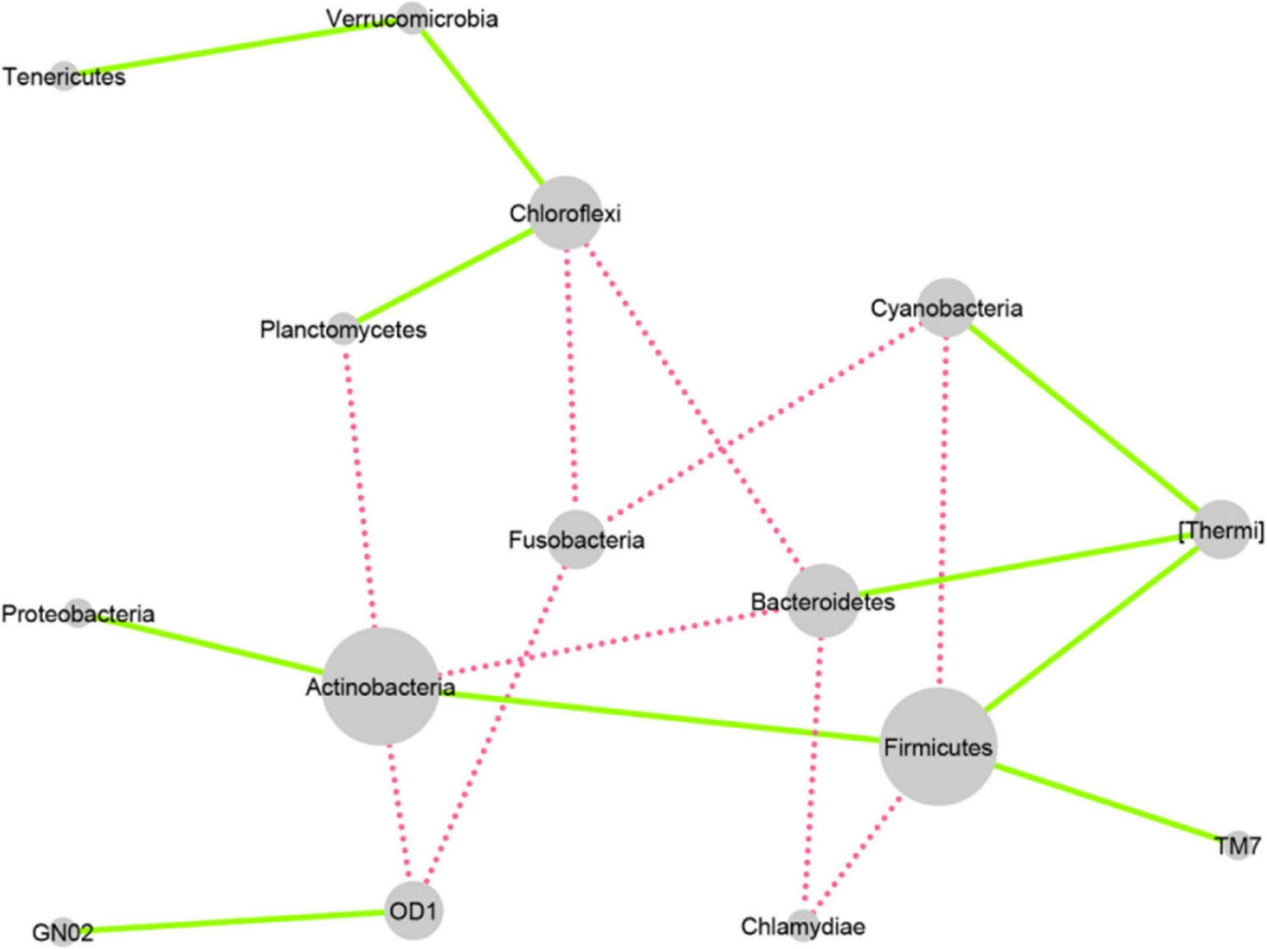
Rewired co-occurrence network at the phylum level. Nodes represent bacterial phyla, and edges represent the statistically significant associations between nodes. The green edges are indicators of gained interactions, and the dashed red edges are indicators of lost interactions in the disease state

**Fig. 10:**
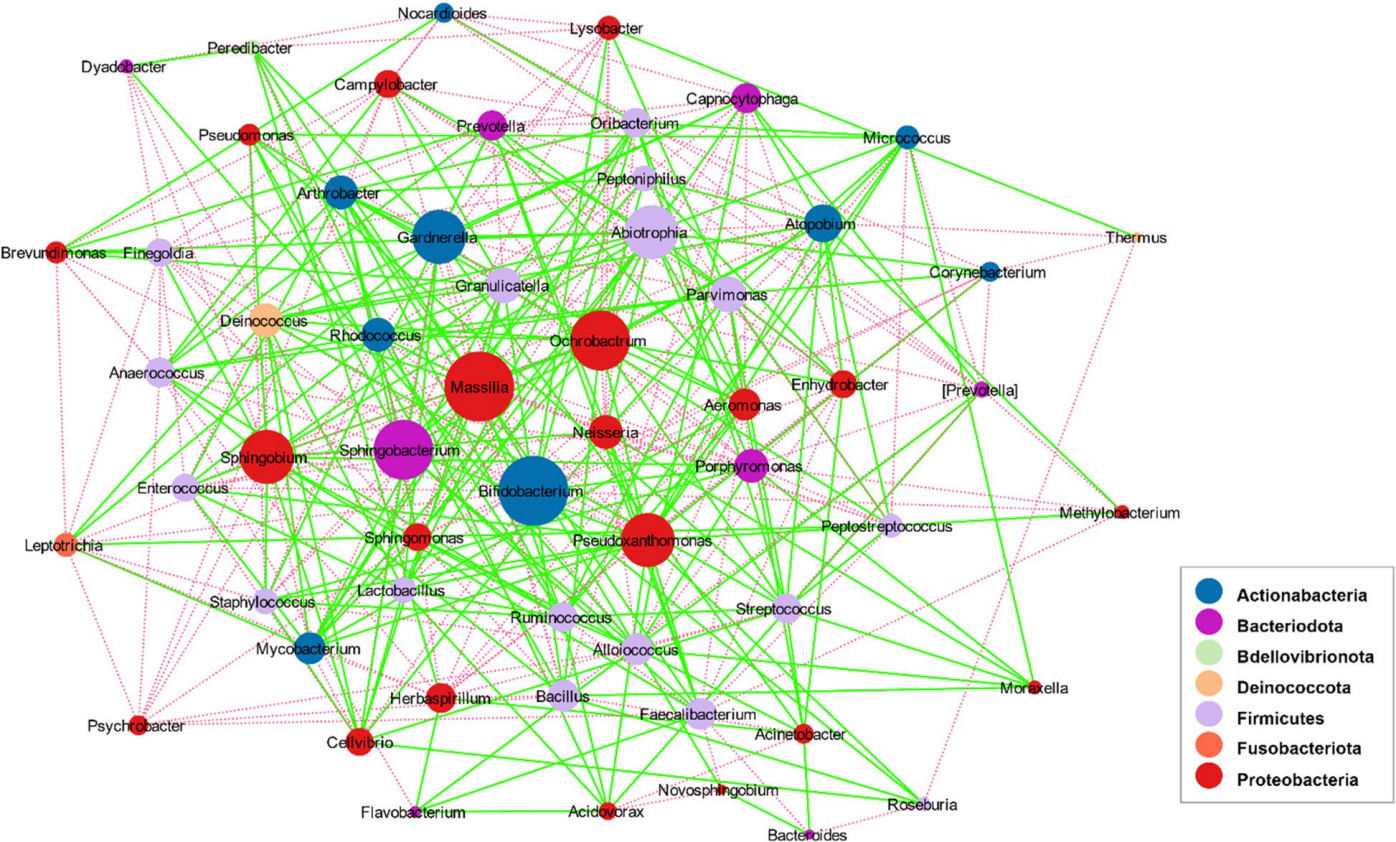
Rewired co-occurrence network at the genus level. Nodes represent bacterial genera, and edges represent the statistically significant associations between nodes. The green edges are indicators of gained interactions, and the dashed red edges are indicators of lost interactions in the disease state

We analyzed differential co-occurrence networks at different taxonomic levels. The rewired network at the phylum level is shown in Fig. 9. The green edges represent the gained interactions, and the dashed red edges represent the lost interactions in the transition from health to the disease state. *Firmicutes* and *Actinobacteria* revealed the highest rewiring degree, in which *Firmicutes* had three gained and two lost interactions. In contrast, *Actinobacteria* had two gained and three lost interactions in lung cancer compared to the normal condition. At the phylum level, we did not detect any changed directions. At the genus level (Fig. 10**)**, we could detect 53 nodes and 373 significant rewired interactions. The green edges represent the gained interactions, and the dashed red edges represent the lost interactions in transitioning from health to disease. *Massilia*, *Bifidobacterium*, *Sphingobacterium*, and *Ochrobactrum* had the highest rewiring degree.

Furthermore, *Massilia*, *Bifidobacterium*, and *Sphingobacterium* revealed the highest closeness in the differential co-occurrence network. We did not detect any changed directions at the genus level. The result obtained at the family level is presented in Additional file 3, in which we could detect 70 nodes and 556 significant rewired interactions. Similarly, the green edges represent the gained interactions, the dashed red edges represent the lost interactions, and the dashed blue edges represent the changed directions in the transition from health to the disease state. The direction of the interactions between two pairs of bacteria (*Methylobacteriaceae* and *Oxalobacteraceae* & *Bacillaceae* and *Bacteroidaceae*) have changed in the disease state compared with the normal condition.

The characteristics and indices of network nodes at the phylum and genus levels were determined. According to the degree index and the obtained results, *Actinobacteria* (with a degree of 5 and betweenness centrality of 0.348), *Firmicutes* (with a degree of 5 and betweenness centrality of 0.239), *Bacteroidetes* (with a degree of 4 and betweenness centrality of 0.204), and *Chloroflexi* (with a degree of 4 and betweenness centrality of 0.309) are the most critical nodes in the phylum-level network, and *Bifidobacterium* (with a degree of 21 and betweenness centrality of 0.0421), *Massilia* (with a degree of 21 and betweenness centrality of 0.0263), *Sphingobacterium* (with a degree of 20 and betweenness centrality of 0.0287), and *Ochrobactrum* (with a degree of 20 and betweenness centrality of 0.0215) are the most critical nodes in the genus-level network. The results of the most important hubs are presented in Table 4. The complete details of the network analysis can be found in Additional file 4.

**Table 4:** Characteristics of the most importantnodes at phylum and genus levels

Level	Degree	Betweenness Centrality	Closeness Centrality	Clustering Coefficient	Neighborhood Connectivity
Genus					
Bifidobacterium	21	0.042161	0.606383	0.22381	13.71429
Massilia	21	0.026314	0.6	0.333333	15.2381
Sphingobacterium	20	0.028788	0.606383	0.284211	15.1
Ochrobactrum	20	0.021511	0.587629	0.294737	15.45
Pseudoxanthomonas	19	0.037796	0.6	0.25731	14.10526
Gardnerella	19	0.028166	0.587629	0.251462	14.47368
Sphingobium	19	0.027283	0.587629	0.274854	14.84211
Abiotrophia	19	0.022107	0.575758	0.263158	15.05263
Atopobium	18	0.03409	0.564356	0.24183	13.77778
Parvimonas	17	0.021138	0.57	0.205882	13.94118
Granulicatella	17	0.018293	0.575758	0.286765	14.94118
Neisseria	16	0.02016	0.57	0.241667	15.375
Porphyromonas	16	0.019818	0.57	0.275	15.125
Rhodococcus	16	0.018362	0.57	0.291667	14.5
Deinococcus	16	0.015364	0.564356	0.383333	15.25
Arthrobacter	16	0.013134	0.553398	0.308333	15.625
Phylum					
Actinobacteria	5	0.348718	0.538462	0	3
Firmicutes	5	0.239927	0.466667	0.1	2.8
Bacteroidetes	4	0.204396	0.518519	0	3.5
Chloroflexi	4	0.309524	0.482759	0	2.75

## Discussion

Although it is worthwhile to identify microbial taxa whose abundance undergoes a significant change in the disease state, it does not provide further information regarding how these differentially abundant taxa interact with other microbial community members. A taxon may not be in abundance, yet it may play a central role in the ecological structure and function of the microbial community for the detection and treatment of respiratory diseases. In the previous study, we evaluated the alterations of the lung microbiome in lung cancer [12]. Here we tried to merge the same datasets to determine the microbiome diversity and conduct a network-based analysis of the lung microbiome in lung cancer.

Our diversity analyses suggested a decreased alpha diversity in lung cancer. Additionally, we did not find a marked difference between the two groups in the taxonomic composition (beta diversity). Different studies reported different alpha and beta diversity results in lung cancer, including increased alpha diversity [15, 16], decreased alpha diversity [17, 18], increased beta diversity [19, 20], decreased beta diversity [17, 21, 22], and no diversity changes [21, 23]. Overall, these results indicate that there is still no definite consensus about the diversity of the lung microbiome composition in lung cancer. One of the advantages of the included studies is that they all used lung biopsies as the best clinical sample for examining the lower respiratory tract microbiome with minimal contamination by the upper respiratory tract microflora. Our previous meta-analysis showed decreased relative abundance of *Actinobacteria* (at the phylum level), *Corynebacteriaceae* and *Halomonadaceae* (at the family level), and *Corynebacterium*, *Lachnoanaerobaculum*, and *Halomonas* (at the genus level) in lung cancer patients. The co-occurrence network analyses here showed that *Actinobacteria* (with a degree of 5 and betweenness centrality of 0.348) and *Firmicutes* (with a degree of 5 and betweenness centrality of 0.239) are the most important nodes in the phylum-level network, and *Bifidobacterium* (with a degree of 21 and betweenness centrality of 0.0421), *Massilia* (with a degree of 21 and betweenness centrality of 0.0263), *Sphingobacterium* (with a degree of 20 and betweenness centrality of 0.0287), and *Ochrobactrum* (with a degree of 20 and betweenness centrality of 0.0215) are the most important nodes in the genus-level network. *Actinobacteria* had a significant role in both our previous meta-analysis and the current study. In our previous study, *Actinobacteria* was found to be differentially abundant between groups, while in the current study, it emerged as the most important node of the network at the phylum level with a degree of 5 and betweenness centrality of 0.348. However, most other taxa exhibited no such relationship. This suggests that other factors beyond differential abundance may play a role in determining the significance of different taxa within the microbiome. Furthermore, we observed that some connections between taxa were lost, and new connections were made in the disease state. Moreover, although in the phylum-level network, *Proteobacteria* was not a key node (with a degree of 1 and no betweenness centrality), most of the important nodes in the genus-level network, including *Massilia*, *Ochrobactrum*, *Pseudoxanthomonas*, and *Sphingobium* belong to this phylum.

The relative abundance of *Firmicutes* and *Bacteroidetes* (specifically the F/B ratio) is considered a criterion for microbiome analyses in several diseases, including obesity [24]. The results of the phylum-level co-occurrence network showed no interaction (either gained or lost) between these two phyla in the disease state. *Actinobacteria*, which co-exists with *Bacteroidetes* and *OD1* in the absence of the disease, lost these interactions and gained two new interactions with *Firmicutes* and *Proteobacteria* in the disease state. Additionally, *Firmicutes* lost its interaction with *Cyanobacteria* and gained a new co-occurrence relationship with *Thermi* and *TM7* phyla in the disease state. Therefore, the increased rate of *TM7* and *Thermi* phyla during lung cancer may be somewhat due to a positive co-occurrence with *Firmicutes*. Analogous to *Firmicutes*, *Actinobacteria* may be regarded as an important network node since these two nodes showed more interactions with other nodes. Our study found *Massilia* and *Bifidobacterium* as the most important nodes in the genus-level co-occurrence network with a degree of 21. However, none of these genera showed a significant difference in abundance between the two groups. A few studies to date have reported *Massilia* in association with lung cancer. A recent study evaluating lung tumor tissue microbiome in lung cancer reported that *Massilia* is more abundant in tumor tissues than in normal tissues [25]. According to the mentioned study, tumorigenesis may provide a microenvironment that promotes the growth of *Massilia. Massilia* and *Sphingobacterium* can degrade polycyclic aromatic hydrocarbons, a well-known carcinogen in cigarette smoke, as a major risk factor in lung cancer patients. In addition, another study also detected *Massilia* in patients with pancreatic cancer [26]. Although the relative abundance of *Cyanobacteria* did not change over the disease state, its interactions in the network changed during the disease, and it gained a new interaction with *Thermi*. *Cyanobacteria* can produce toxin metabolites and increase inflammation in the lung [27]. However, the results obtained in this study may be different, and *Cyanobacteria* could potentially play alternative roles as an effective node. Although the relative frequency of the phylum *Verrucomicrobia* (including *Akkermansia* spp.) did not significantly change (Table 2), it formed co-occurrence interactions with both *Chloroflexi* and *Tenericutes* in the phylum-level network. *Akkermansia* has an effective role in reducing inflammation by producing short-chain fatty acids through the breakdown of mucin. *Verrucomicrobia* and *Chlamydiae* are sister phyla that differ in conserved signature indels (CSIs). Interestingly, new connections have been established in the network between *Verrucomicrobia* and two other phyla (*Tenericutes* and *Chloroflexi*). However, in the case of *Chlamydiae*, its interactions with *Firmicutes* and *Bacteroidetes* have been lost. We could not find any significant difference in the relative abundance of *Veillonella*, *Streptococcus*, and *Prevotella* between cases and controls, however, *Streptococcus* and *Prevotella* were found to be important nodes as indicated in the genus-level network. *OD1* and *TM7* are among a large group of bacteria known as “Candidate Phyla Radiation” (CPR) bacteria [28]. *OD1* has been recently found in cystic fibrosis, indicating a potentially important role in causing microbiome dysbiosis in the respiratory system [29, 30]. *TM7* is another CPR phylum with a demonstrated epibiotic parasitic lifestyle. A few species of *TM7* have been found to be in association with *Actinobacteria *[31, 32]. In the phylum-level co-occurrence network, *TM7* (with a degree of 1) interacted indirectly through *Firmicutes* with *Actinobacteria*, suggesting its probable feeding on *Actinobacteria* during the disease state. It is still unclear what exact role these phyla play in the disease state. Regarding the poor degree of *TM7*, it may be a driver node, which needs further research to be answered. In addition, our study identified no family or genus of *OD1*, which may be due to the unknown identity of most members of this phylum [31]*.*

We found a significant difference in the relative abundance of *Bacteroidetes* (as an important node in the phylum-level network with a degree of 4 and betweenness centrality of 0.204) between health and disease states, as indicated in other lung diseases such as cystic fibrosis [10]. Producing anaerobic fermentative acidic products such as short-chain fatty acids can result in airway inflammation, tissue damage, and airway remodeling [10]. Although no significant difference was seen in the relative abundance of *Comamonadaceae*, it was found in a distinct module along with *Rhodocyclaceae* as the result of the DBSCAN clustering algorithm suggested. They are closely related to acetate-utilizing denitrifiers families that are probably involved in denitrification. In another study, the *Comamonadaceae* family has been associated with airway hyper-reactivity in asthma patients [33].

The clustering of microbiome taxa is as important as differential abundance and diversity analyses. Accordingly, several microbiome studies have used the DBSCAN clustering method due to some of its features [34]. Specifically, the DBSCAN algorithm requires two parameters: 1) the radius of the circle, which specifies the neighborhood or region to check the density, and 2) the minimum number of features or data points required inside that circle. Since metagenomics data are considered high-dimensional with multiple noise and outliers, the abovementioned features make this algorithm particularly suitable for microbiome data analysis. Unfortunately, we could not sufficiently interpret the results obtained by DBSCAN, which may be due to the lack of annotation servers to help us provide further information about these modules. If such servers are enriched, a better understanding of the metabolic commonalities of these bacteria and their association with lung cancer could be acquired. As mentioned earlier, one of the advantages of this study is that it is based on the lung biopsy specimen, which best represents the respiratory microbiome. However, the study is limited by the lack of information on patients’ metadata. The importance of this limitation lies in the fact that distinguishing between the two subtypes of NSCLC could have affected the final picture of the lung microbial community in the disease state since the composition of the lung microbiome may be unique in each of these subtypes. In conclusion, we tried to determine how interactions of the lung microbiome members change in the disease state and examine the respiratory microbiome using a combination of integrative and network-based approaches. As a valuable finding, we observed that the differentially abundant taxa did not necessarily play a central role in the co-occurrence network. Several genera (e.g., *Bifidobacterium* and *Massilia*) appeared in the network with key roles, while their relative abundance was not significantly different in health and disease states. These findings may provide new insights into the identification of key members of the lung microbial community in the context of lung cancer, underscoring the importance of further research to advance the understanding of their functions and interactions.

## Conclusions

The present study had two primary aims. Firstly, we integrated several metagenomic studies on lung cancer to achieve more robust results regarding changes in the relative abundance of taxa and diversity indices of the lung microbiome content in patients with NSCLC. We adopted a standardized approach whereby raw sequence data in each study were processed into relative abundance data independently before integrating them. This method was preferred due to its ability to mitigate the impact of potential technical variations across studies while ensuring the comparability of different datasets. Our analyses showed significant differences in the relative abundance of bacterial taxa at different taxonomic levels and suggested reduced alpha diversity in the tumor microbiome compared to normal controls. However, considering the inconsistencies across microbiome studies, there is still no conclusive evidence regarding the diversity of the lung microbiome in lung cancer. Secondly, we attempted to evaluate the co-existing relationships among the community members. The findings of this study suggest that while *Proteobacteria* did not appear to play a significant role in the broader network of bacterial phyla, several important nodes in the genus-level network (e.g., *Massilia*, *Ochrobactrum*, *Pseudoxanthomonas*, and *Sphingobium*) belong to this phylum. This finding highlights the need to examine these specific bacterial genera more closely to better understand their potential role in the pathogenesis of lung cancer. Furthermore, it is important to note that certain taxa that exhibit no statistically significant differences between groups should not be dismissed from further investigation, as they may hold central roles within the network. For instance, *Actinobacteria* and its genus *Bifidobacterium* have been found to play crucial roles in the co-occurrence networks despite not showing significant group differences. Interestingly, of the four most important microbial nodes at the genus level (e.g., *Bifidobacterium*, *Massilia*, *Sphingobacterium*, and *Ochrobactrum*), none were significantly different in terms of relative abundance between the two groups but appeared to have vital roles in the network. In addition, we identified four distinct clusters of closely related bacterial families in lung cancer patients. Further research could explore how the metabolic commonalities of these clustered bacteria are associated with lung cancer and whether distinct microbiome compositions are associated with specific subtypes or stages of lung cancer. These findings may provide insights into identifying the key members of the lung microbial community that contribute to the pathogenesis of the disease.

## Methods

### Systematic literature search

A systematic literature search of PubMed was conducted to identify metagenomic studies related to lung cancer. The combination of the following keywords was used for the systematic search: ((“Lung Cancer” [Title/Abstract]) OR (“Pulmonary Neoplasm” [Title/Abstract])) AND ((“Microbiome” [Title/Abstract]) OR (“Metagenom*” [Title/Abstract])), which was last finalized on January 8, 2021. High-throughput sequencing (aka next-generation sequencing (NGS)) repositories, including Sequence Read Archive (SRA, NCBI), Gene Expression Omnibus (GEO, NCBI), ArrayExpress (EMBL-EBI), and MGnify (EMBL-EBI) were also surveyed. The full text of potentially eligible articles was assessed after initial screening and excluding articles unrelated to the topic. We excluded studies that relied on clinical respiratory samples other than lung biopsy (such as sputum, bronchial washings, or bronchoalveolar lavage samples) or lacked an accession number, as well as studies that implemented a clinical intervention or lacked accompanying clinical information. Finally, five case–control studies with available raw sequence data were selected for quantitative analysis. During the process of identifying common taxa from the datasets to reconstruct the co-occurrence networks, we chose to exclude 14 samples (as the smallest dataset with the accession number “PRJNA472758”) in the screening step and proceed with the analysis of the remaining four datasets, which comprise a total of 835 biopsy samples.

Regarding integrating these datasets, it is worth noting that although these datasets were not wholly homogeneous, we combined them based on specific criteria such as similar platforms and 16S rRNA PCR protocols. As is common in microbiome studies, we utilized relative frequency data instead of raw data. This approach allowed us to make comparisons between variables within and across datasets.

### Quality control and data pre-processing

The 16S rRNA sequence data were processed using QIIME 2 (version 2020.6) pipeline [35]. Before running the QIIME scripts, the quality of sequences was visualized using the FastQC tool [36], which provides quality control checks on raw sequence data and facilitates the choice of filtering parameters (e.g., trimming and truncating parameters). All values assigned to the filtering parameters can be found in Additional file 5. Once the QC checks were completed, demultiplexed paired-end reads in FASTQ format were imported into QIIME. The reads were then denoised into amplicon sequence variants (ASVs). The denoising approach was adopted since it has a number of advantages over the clustering approach and OTU picking process [37]. The DADA2 method [38] was employed for the denoising step to remove non-biological or low-quality reads, correct sequencing errors, and join forward and reverse reads. This procedure resulted in a matrix (aka feature table) representing the number of times each feature (ASV) is observed in each sample. We completed the taxonomy classification step by comparing our ASVs to the Greengenes reference database (version 13.8) [39] with known taxonomies to identify the bacterial taxa in samples. The whole process was repeated for each included dataset. Finally, the four resulting feature tables were merged into a single table based on which analyses were carried out.

### Estimation of diversity indices

Shannon and Simpson diversity indices were used to calculate the alpha diversity. The estimated indices were then visualized using Boxplots to make comparisons between groups. A Wilcoxon Rank Sum Test was used to determine whether the two groups’ differences were statistically significant. To assess whether or not two communities are different in terms of overall taxonomic composition, we measured beta diversity based on Bray–Curtis dissimilarity, and the resulting dissimilarity matrix was then plotted using ordination techniques. Specifically, principal coordinate analysis (PCoA) was adopted to visualize the microbiome variation between samples. To evaluate whether the sample type has a significant effect on overall lung microbiome composition, a permutational multivariate analysis of variance (PERMANOVA) was used. Beta-dispersion was quantified to measure variance homogeneity and then subjected to analysis of variance (ANOVA) to determine statistical significance. Significance levels were set at 5%, and all analyses were carried out using R. In particular, the vegan package [40] was used for diversity quantification, ggplot2 [41] for data visualization, and stats [42] for statistical hypothesis testing. In order to comprehensively investigate alpha and beta diversity, all relevant analyses were undertaken on the merged dataset and the individual datasets of each study.

### Differential abundance analysis and clustering

To check the normality of the data, we first conducted the Shapiro–Wilk test, and as the data were not normally distributed., the Wilcoxon Rank Sum Test was used to make a comparison between cases and controls at phylum, family, and genus taxonomic levels. Benjamini-Hochberg (BH) method, which controls the false discovery rate (FDR), was used to adjust for multiple comparisons. To detect the modules at family and genus levels, we implemented the density-based spatial clustering of applications with noise (DBSCAN) clustering method [43] using the dbscan R package (v1.1–5). The Euclidean distance was used for clustering.

### Construction of Co-occurrence networks

The differential networks represent the dynamic changes between two conditions, such as interactions between taxa in transition from normal condition to disease state. In this study, to construct the differential co-occurrence networks at different taxonomic levels, Pearson correlation coefficients (PCC) were computed for bacterial taxa. The analyses were performed at phylum, family, and genus levels. The co-occurrence networks were constructed for health and disease states, respectively. The statistically significant interactions/PCCs were selected for the final networks, and the non-significant PCCs were transformed to zero.

**Fig. 11:**
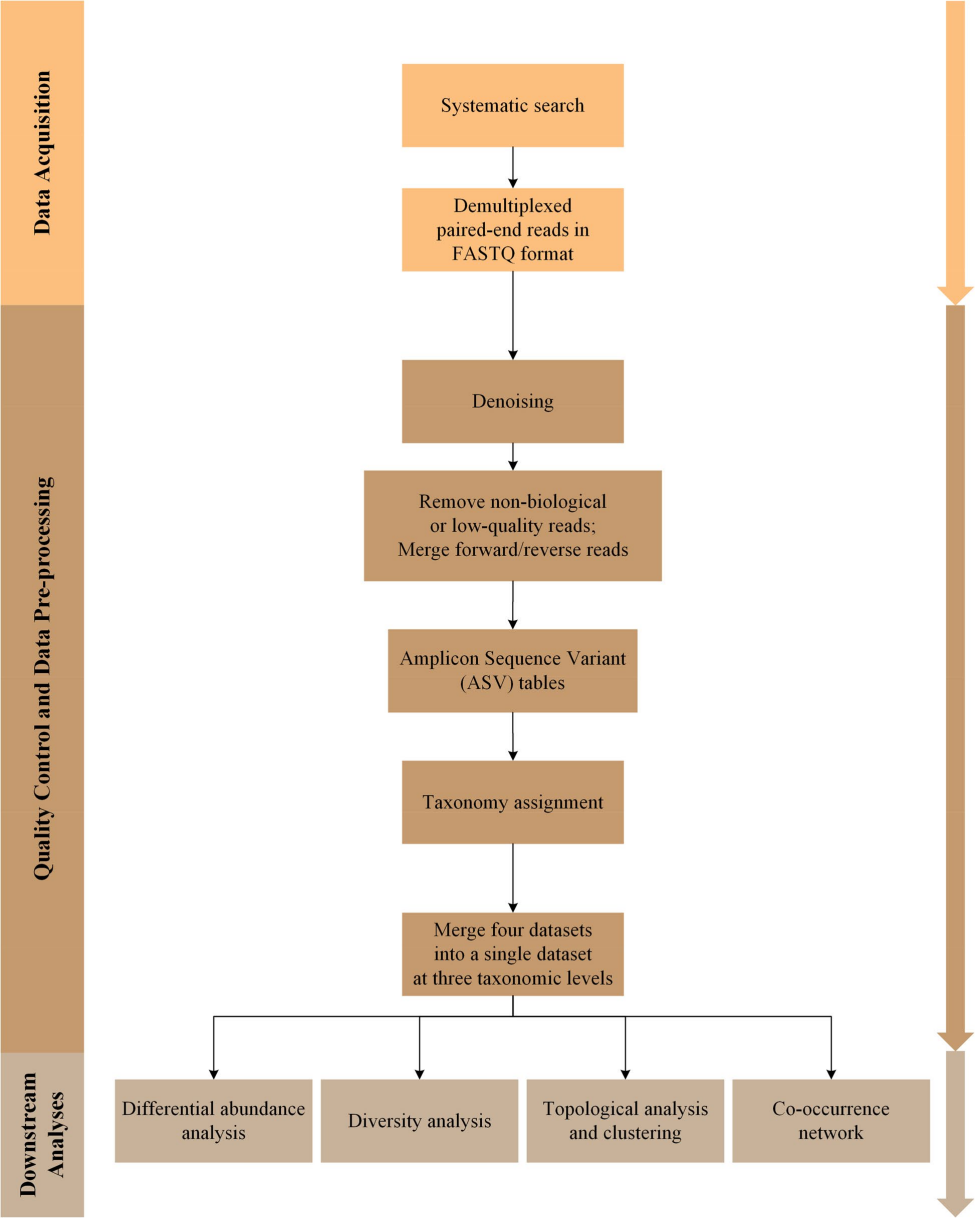
An overall workflow including data acquisition and preparation steps followed by downstream analyses

Moreover, the differential network was reconstructed at each taxonomic level to detect the changed interactions (rewiring) in the transition from health to disease state. In the differential network, if an interaction between taxa emerged in cancer while it did not exist in the normal condition, we called it a “gained interaction”. In contrast, if an interaction disappeared in cancer while it existed in the normal condition, we called it a “lost interaction”. Finally, if the sign of interactions were changed between normal and cancer, we called it a “changed direction”. Important nodes in the network were determined according to the degree index. To this end, a cut-off of greater than or equal to 4 was considered for the phylum-level network, and a cut-off of greater than 15 was considered for the genus-level network. All network construction computations were implemented in R, and the networks were visualized using Cytoscape software [44]. An overall workflow is presented in Fig. 11.

## Supplementary Information

**Additional file 1. **Scaled relative frequency of shared taxa across different datasets at phylum and family levels.

**Additional file 2. **Diversity analyses in individual datasets.

**Additional file 3. **Rewired co-occurrence network at the family level.

**Additional file 4. **Characteristics of co-occurrence networks at phylum and genus levels.

**Additional file 5. **Values assigned to filtering parameters used in the denoising step in QIIME.

## Data Availability

The datasets analyzed for this study can be found in the NCBI SRA repository (https://www.ncbi.nlm.nih.gov/sra). The accession numbers are as follows: PRJNA472758, PRJNA624822, PRJNA303190, PRJNA327258, and PRJNA647170. R codes are available upon request.
