# Supplementary material for: Exploring Co-occurrence patterns and microbial diversity in the lung microbiome of patients with non-small cell lung cancer
Source: BMC Microbiol. 2023 Jul 11;23:182. doi: 10.1186/s12866-023-02931-9 (PMC10334658; doi:10.1186/s12866-023-02931-9)
Supplement: Supplementary file 1 — Additional file 1. Scaled relative frequency of shared taxa across different datasets at phylum and family levels. [file 12866_2023_2931_MOESM1_ESM.docx]

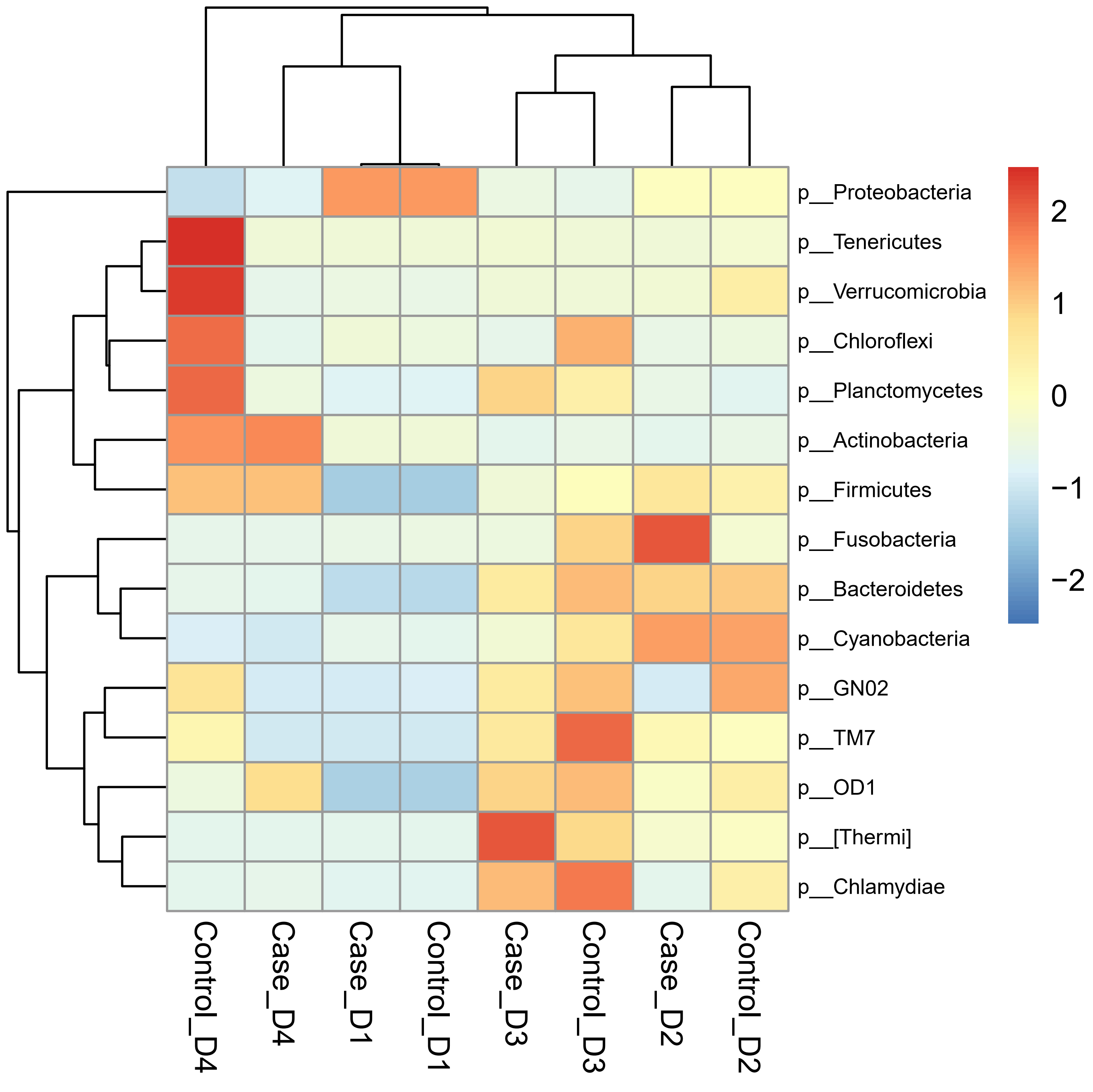


Fig. S1 Scaled relative frequency of shared phyla across different datasets. The heatmap displays the variation in the microbiome composition at the phylum level. The average relative frequency of the shared phyla has been shown. D1: Dataset 1, D2: Dataset 2, D3: Dataset 3, D4: Dataset 4.


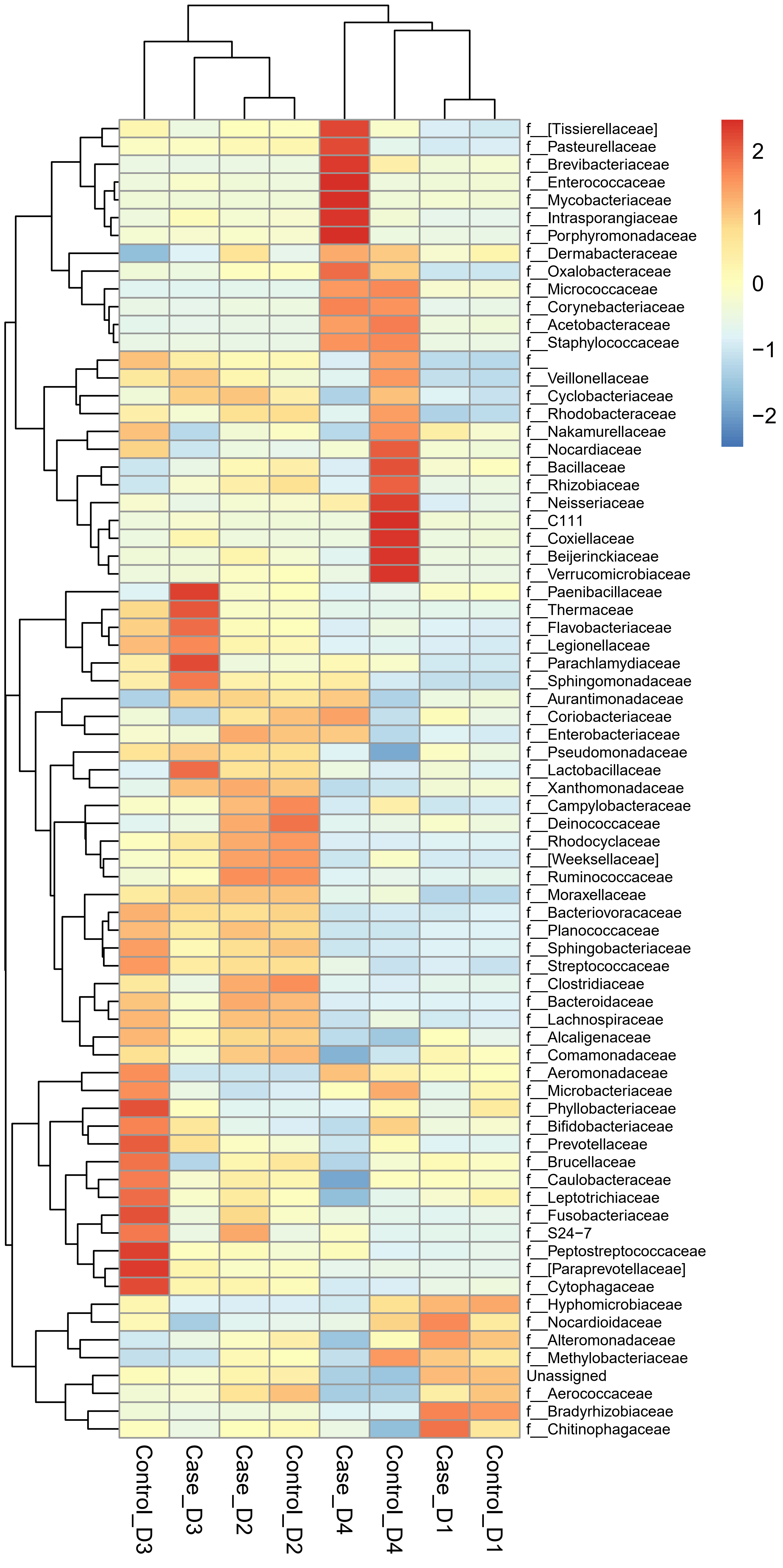


Fig. S2 Scaled relative frequency of shared families across different datasets. The heatmap displays the variation in the microbiome composition at the family level. The average relative frequency of the shared families has been shown. D1: Dataset 1, D2: Dataset 2, D3: Dataset 3, D4: Dataset 4.
