# Supplementary material for: Exploring Co-occurrence patterns and microbial diversity in the lung microbiome of patients with non-small cell lung cancer
Source: BMC Microbiol. 2023 Jul 11;23:182. doi: 10.1186/s12866-023-02931-9 (PMC10334658; doi:10.1186/s12866-023-02931-9)
Supplement: Supplementary file 2 — Additional file 2. Diversity analyses in individual datasets. [file 12866_2023_2931_MOESM2_ESM.docx]

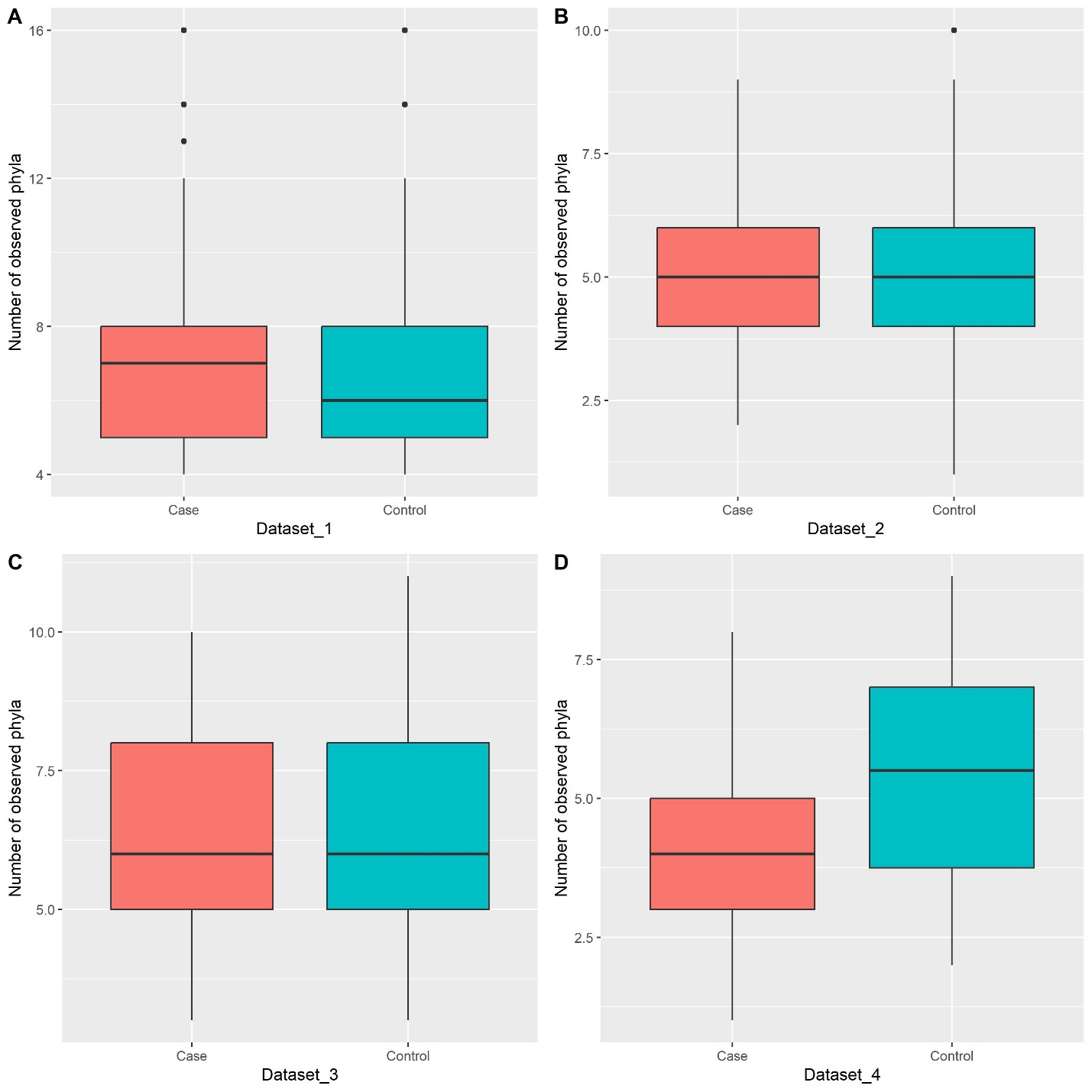


Fig. S3 Alpha diversity at phylum level in individual datasets. Boxplots summarize estimated alpha diversity based on the number of observed phyla (richness) within each group and show differences between cases and controls. The differences between the two groups were tested using the Wilcoxon Rank Sum Test the results of which were not statistically significant. Case: lung tumor tissues, Control: tumor-adjacent normal tissues.


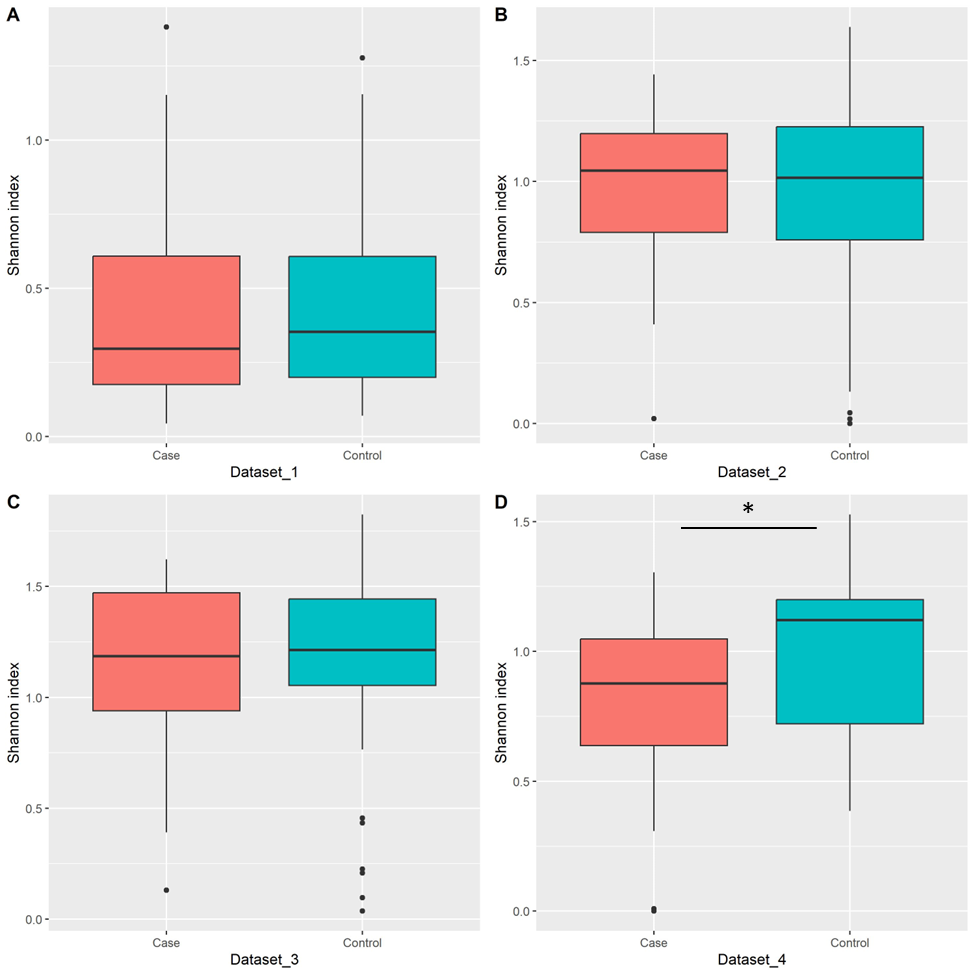


Fig. S4 Alpha diversity at phylum level in individual datasets. Boxplots summarize estimated alpha diversity based on Shannon diversity index (aka Shannon-Wiener index) within each group and show differences between cases and controls. Asterisk represents a significant result from the Wilcoxon Rank Sum Test. Case: lung tumor tissues, Control: tumor-adjacent normal tissues, *p < 0.05.


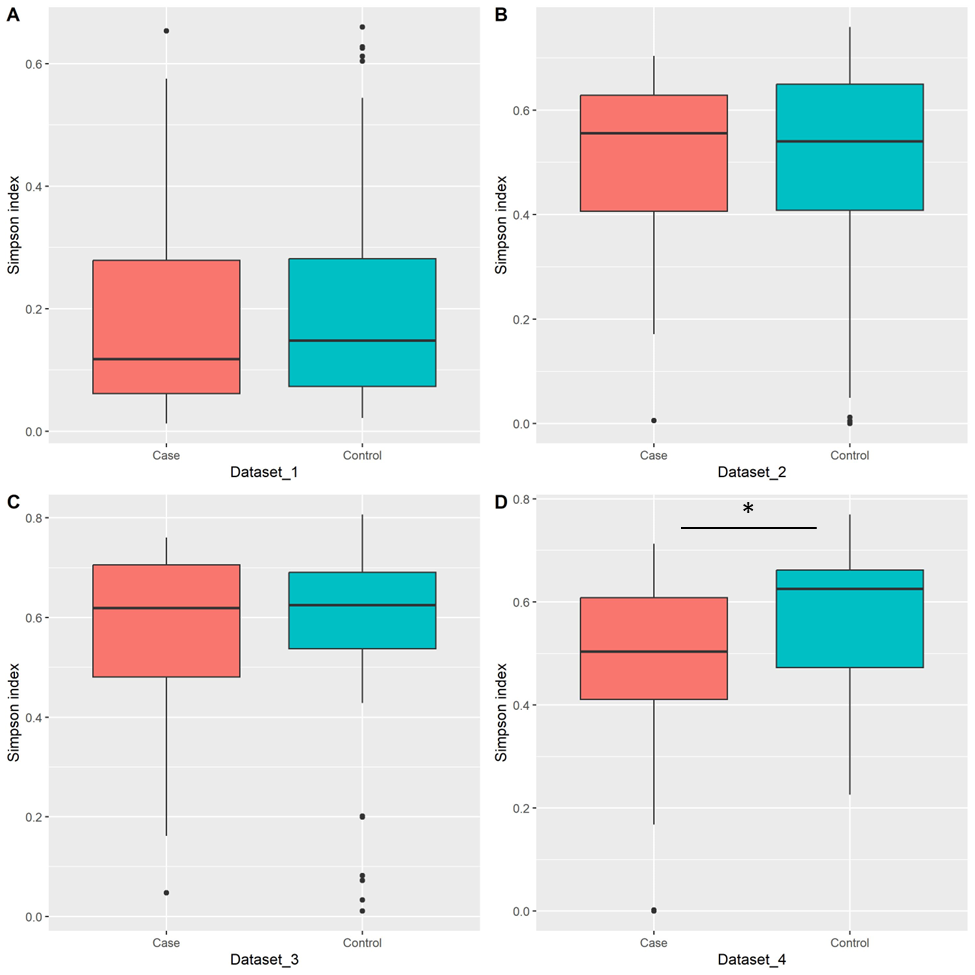


Fig. S5 Alpha diversity at phylum level in individual datasets. Boxplots summarize estimated alpha diversity based on Simpson index within each group and show differences between cases and controls. Asterisk represents a significant result from the Wilcoxon Rank Sum Test. Case: lung tumor tissues, Control: tumor-adjacent normal tissues, *p < 0.05.


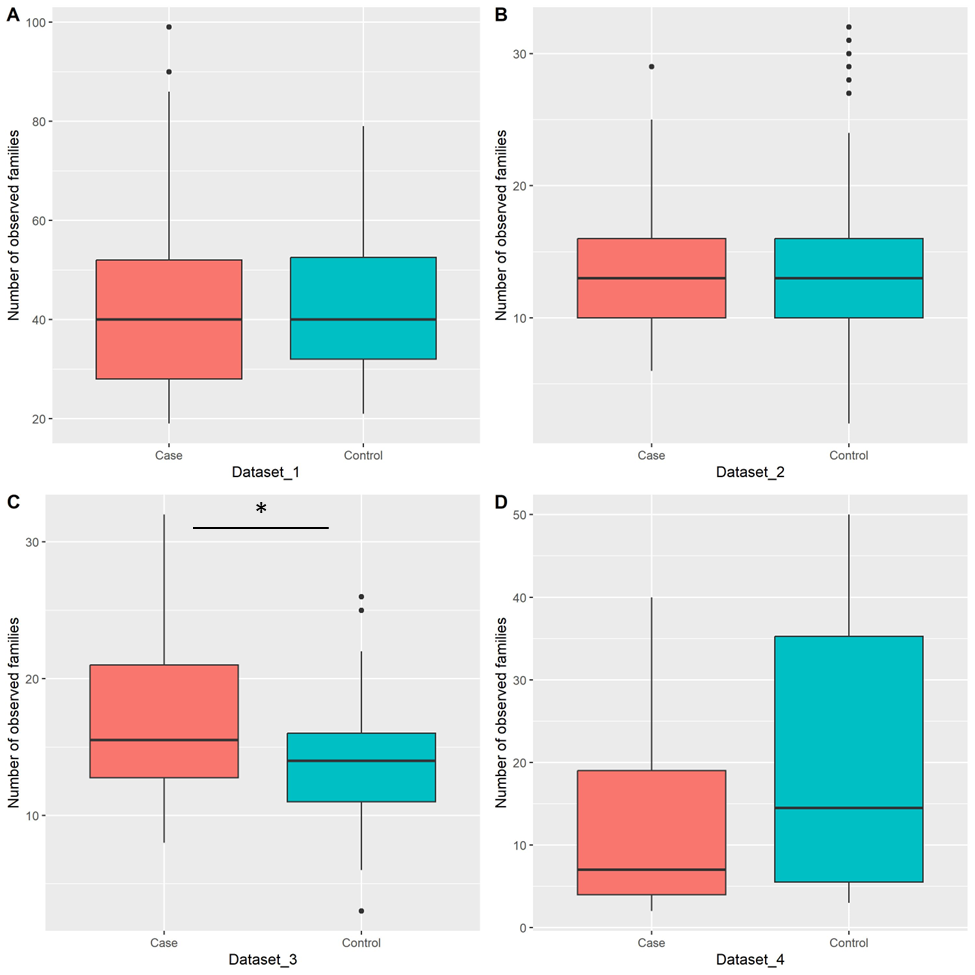


Fig. S6 Alpha diversity at family level in individual datasets. Boxplots summarize estimated alpha diversity based on the number of observed families (richness) within each group and show differences between cases and controls. Asterisk represents a significant result from the Wilcoxon Rank Sum Test. Case: lung tumor tissues, Control: tumor-adjacent normal tissues, *p < 0.05.


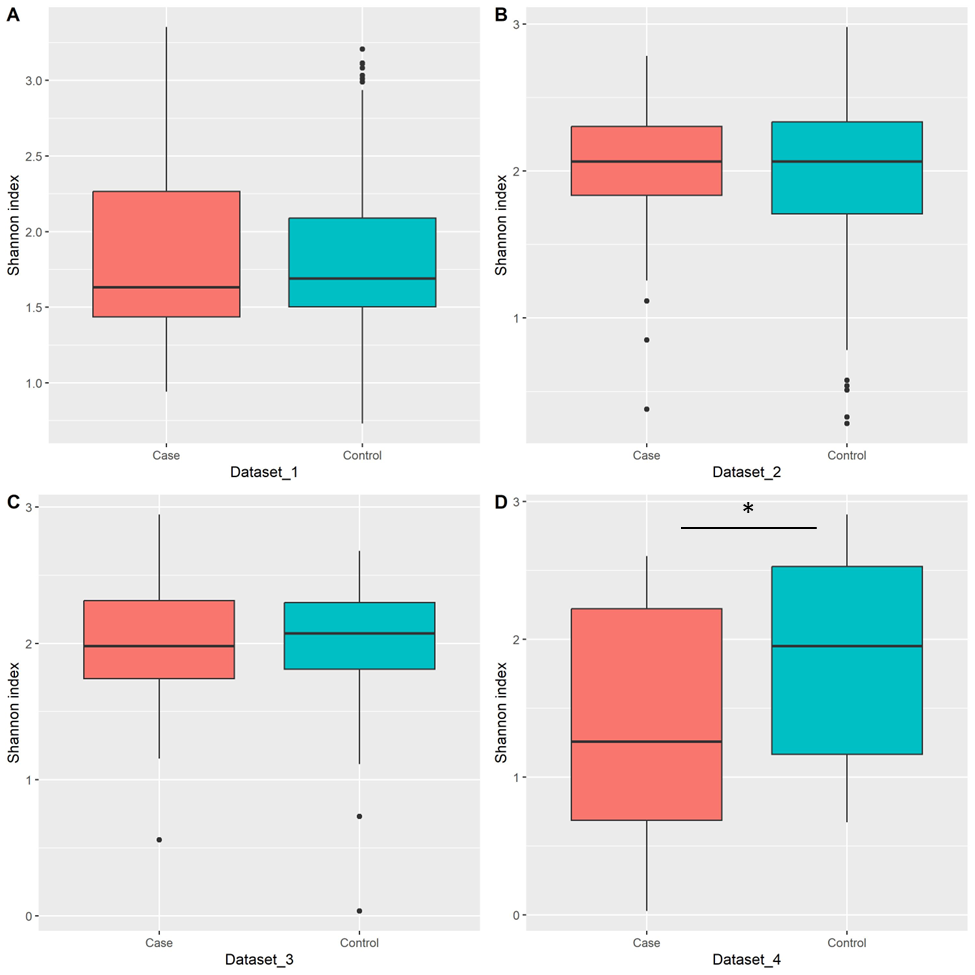


Fig. S7 Alpha diversity at family level in individual datasets. Boxplots summarize estimated alpha diversity based on Shannon diversity index (aka Shannon-Wiener index) within each group and show differences between cases and controls. Asterisk represents a significant result from the Wilcoxon Rank Sum Test. Case: lung tumor tissues, Control: tumor-adjacent normal tissues, *p < 0.05.


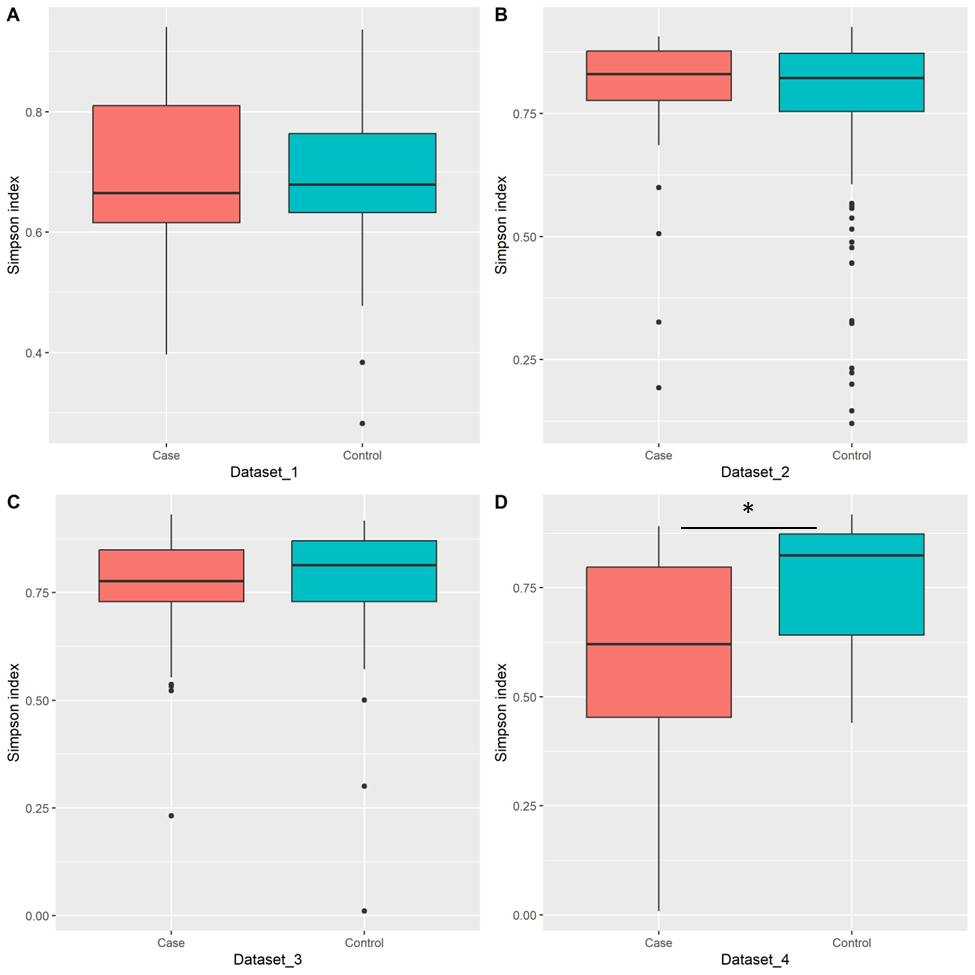


Fig. S8 Alpha diversity at family level in individual datasets. Boxplots summarize estimated alpha diversity based on Simpson index within each group and show differences between cases and controls. Asterisk represents a significant result from the Wilcoxon Rank Sum Test. Case: lung tumor tissues, Control: tumor-adjacent normal tissues, *p < 0.05.


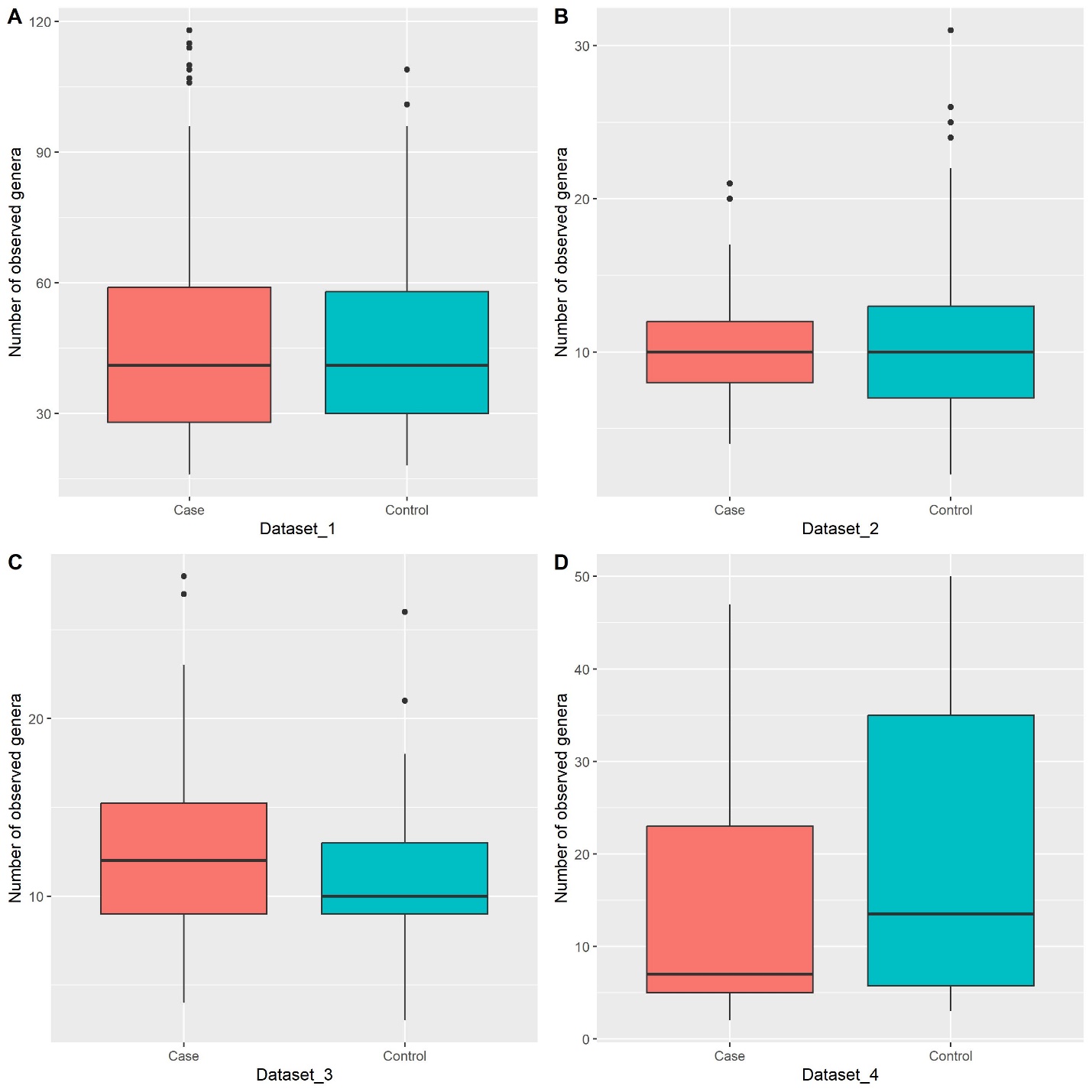


Fig. S9 Alpha diversity at genus level in individual datasets. Boxplots summarize estimated alpha diversity based on the number of observed genera (richness) within each group and show differences between cases and controls. The differences between the two groups were tested using the Wilcoxon Rank Sum Test the results of which were not statistically significant. Case: lung tumor tissues, Control: tumor-adjacent normal tissues.


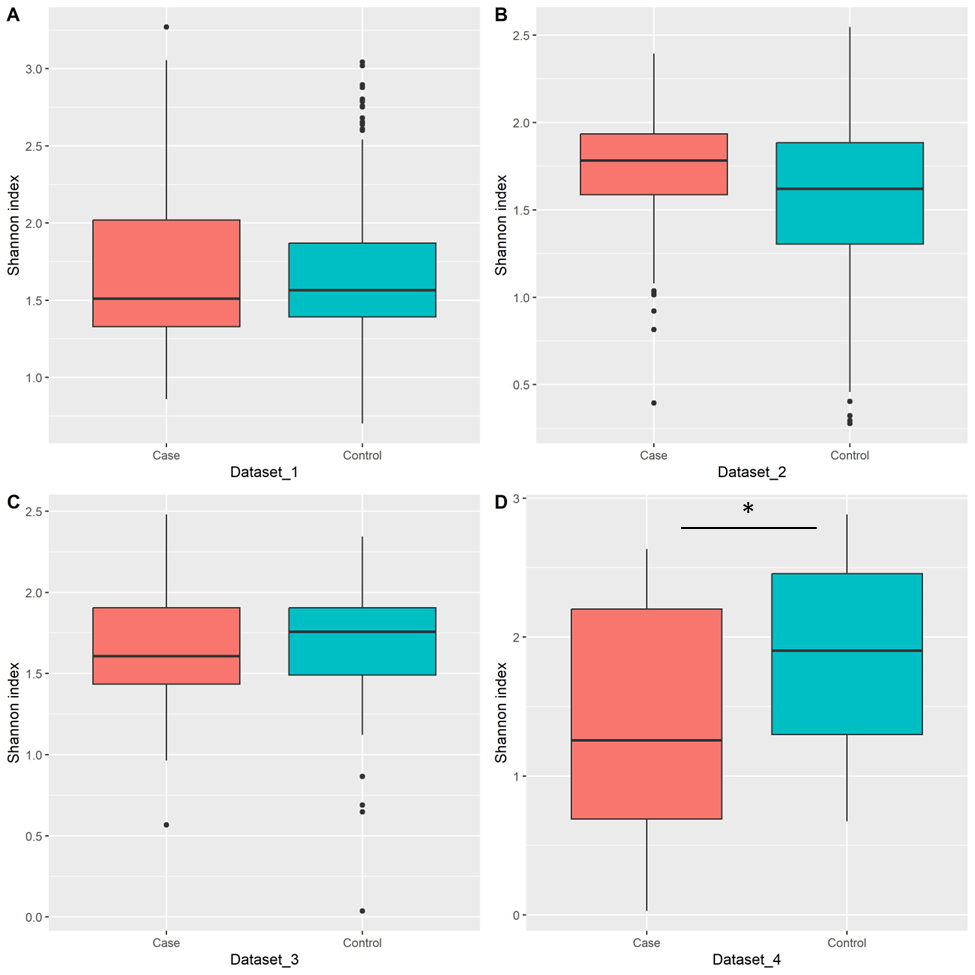


Fig. S10 Alpha diversity at genus level in individual datasets. Boxplots summarize estimated alpha diversity based on Shannon diversity index (aka Shannon-Wiener index) within each group and show differences between cases and controls. Asterisk represents a significant result from the Wilcoxon Rank Sum Test. Case: lung tumor tissues, Control: tumor-adjacent normal tissues, *p < 0.05.


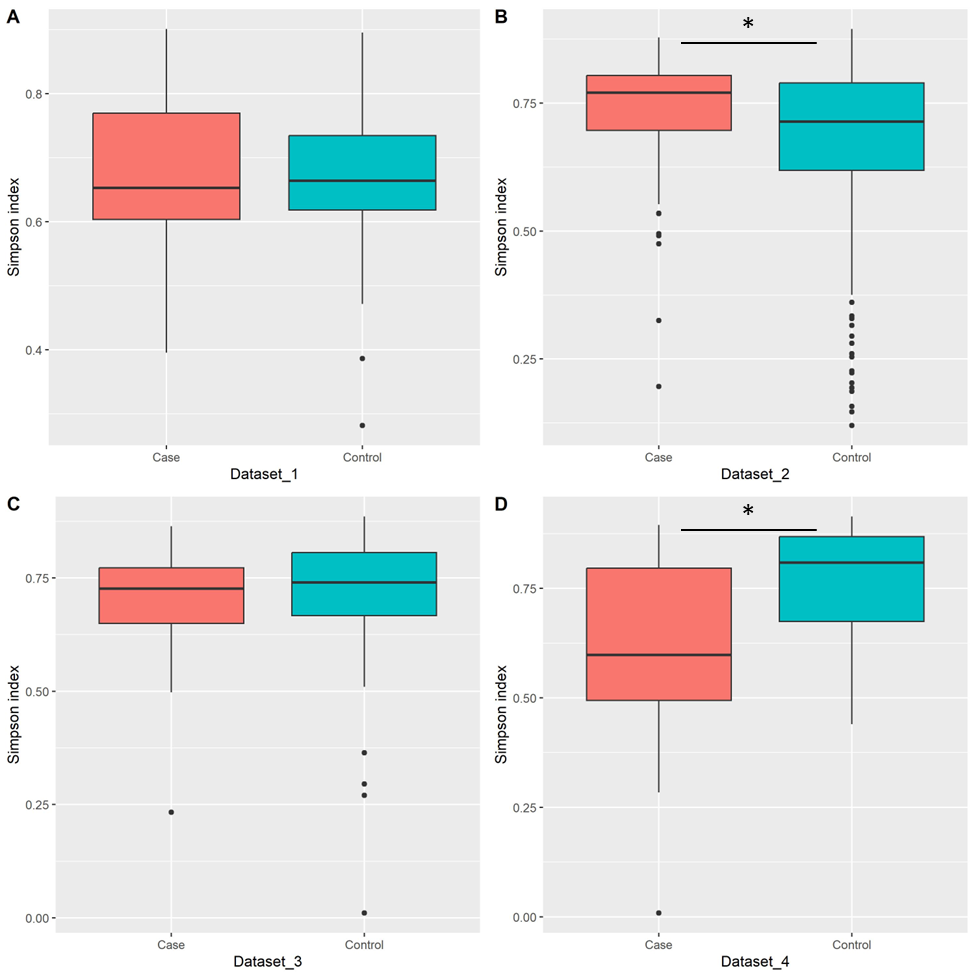


Fig. S11 Alpha diversity at genus level in individual datasets. Boxplots summarize estimated alpha diversity based on Simpson index within each group and show differences between cases and controls. Asterisks represent a significant result from the Wilcoxon Rank Sum Test. Case: lung tumor tissues, Control: tumor-adjacent normal tissues, *p < 0.05.


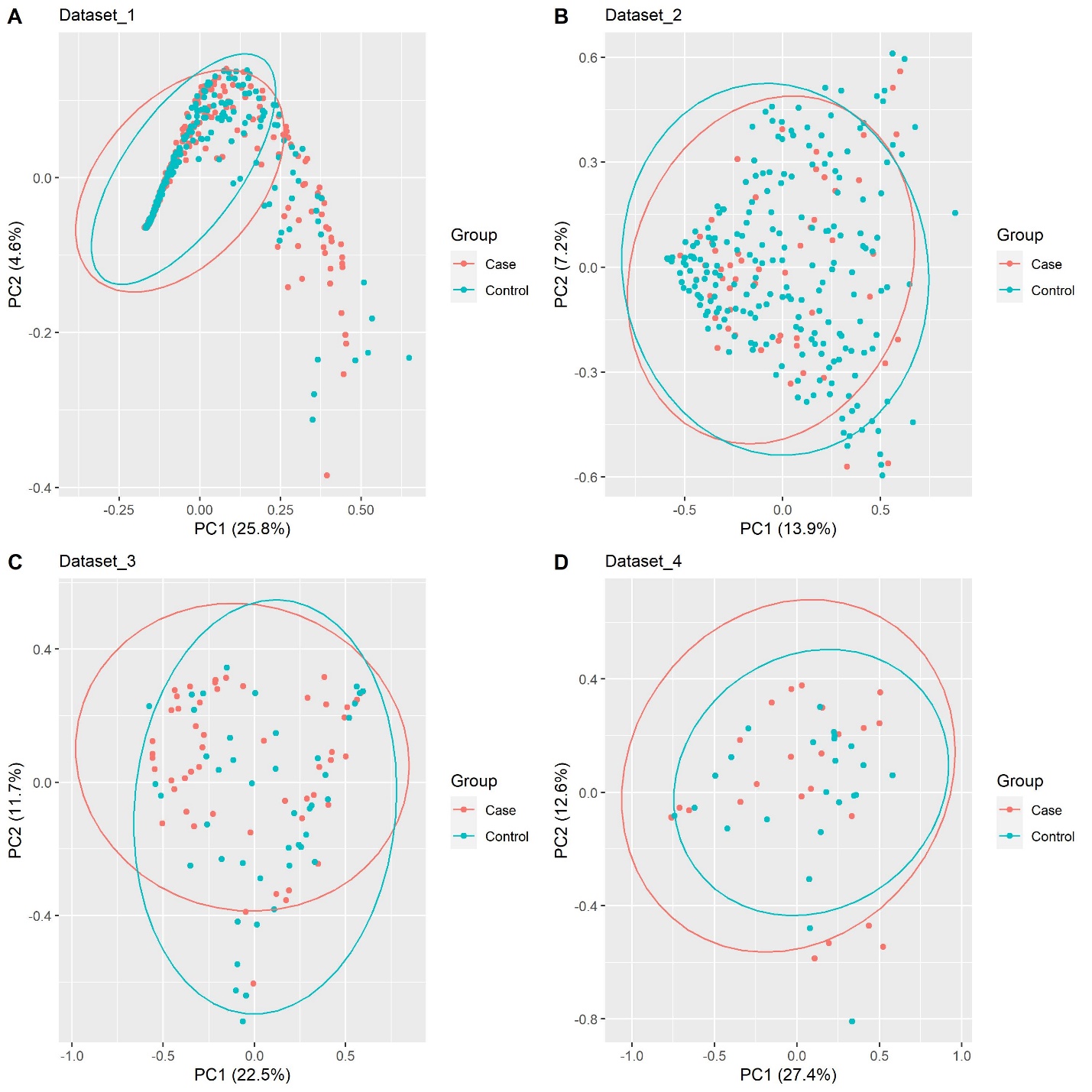


Fig. S12 Beta diversity at phylum level in individual datasets. PCoA plots are based on Bray-Curtis dissimilarity which show the differences in the composition of the lung microbiome between cases and controls. Each dot represents the microbiome of a sample. The differences between the two groups were tested using PERMANOVA the results of which were not statistically significant. Case: lung tumor tissues, Control: tumor-adjacent normal tissues.


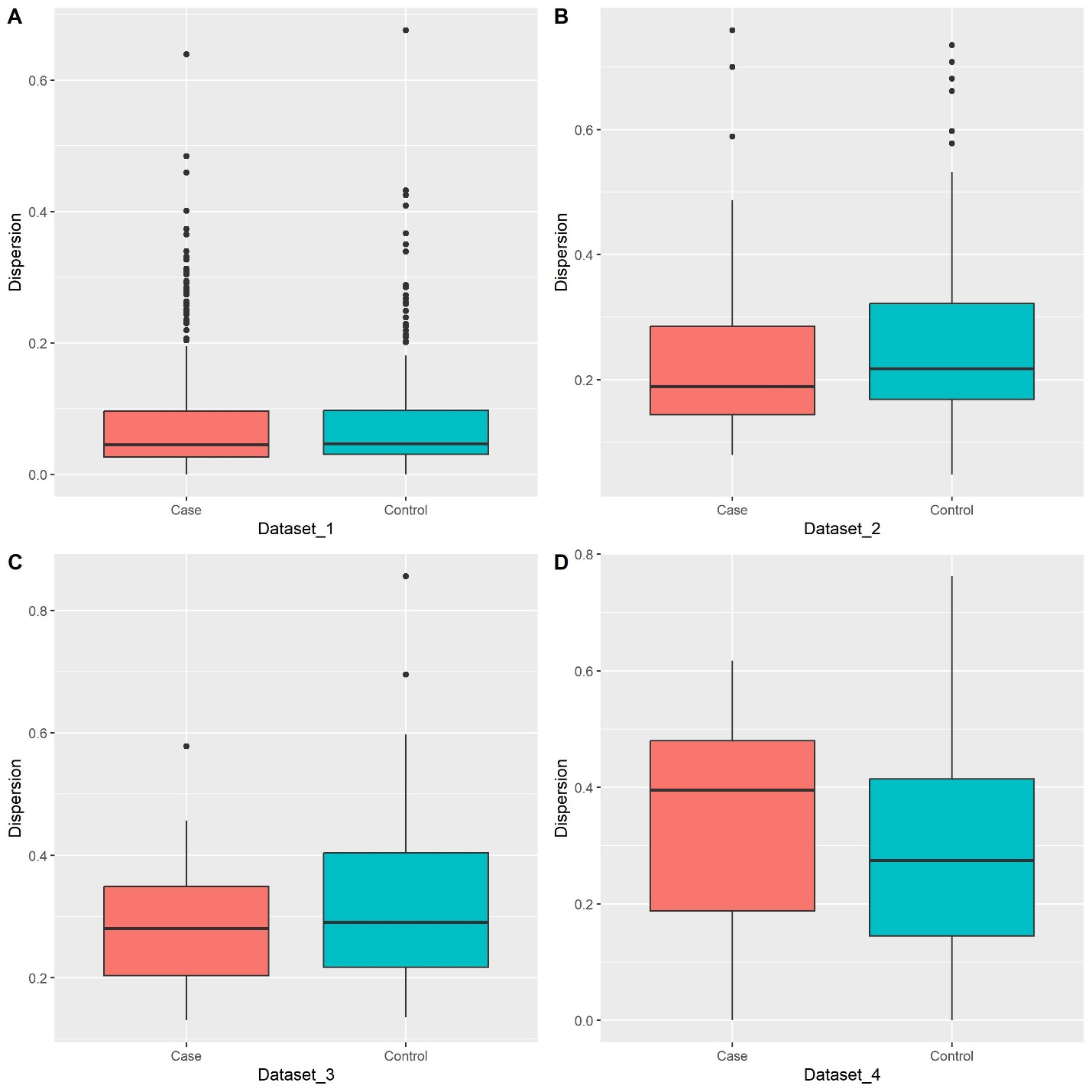


Fig. S13 Beta dispersion at phylum level in individual datasets. Boxplots show dispersion within and between groups. The differences between the two groups were tested using ANOVA the results of which were not statistically significant. Case: lung tumor tissues, Control: tumor-adjacent normal tissues.


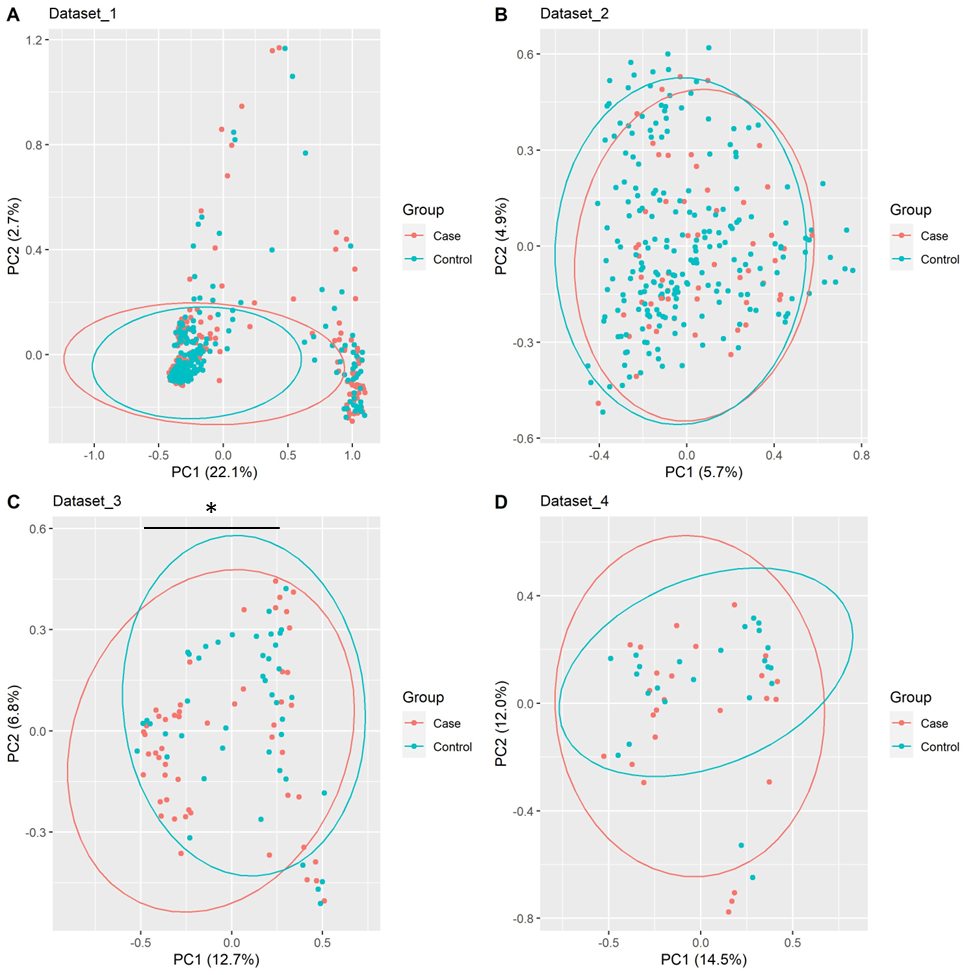


Fig. S14 Beta diversity at family level in individual datasets. PCoA plots are based on Bray-Curtis dissimilarity which show the differences in the composition of the lung microbiome between cases and controls. Each dot represents the microbiome of a sample. Asterisk indicates a significant result from PERMANOVA. Case: lung tumor tissues, Control: tumor-adjacent normal tissues, *p < 0.05.


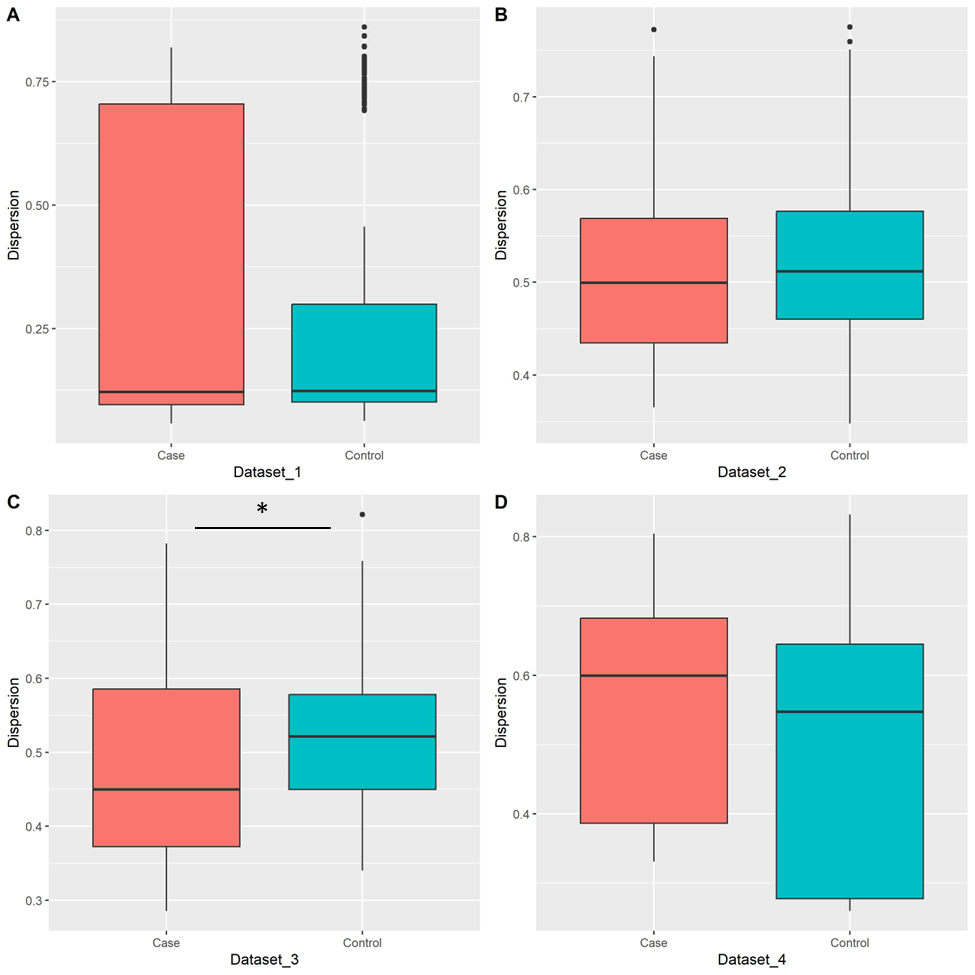


Fig. S15 Beta dispersion at family level in individual datasets. Boxplots show dispersion within and between groups. Asterisk represents a significant result from ANOVA. Case: lung tumor tissues, Control: tumor-adjacent normal tissues, *p < 0.05.


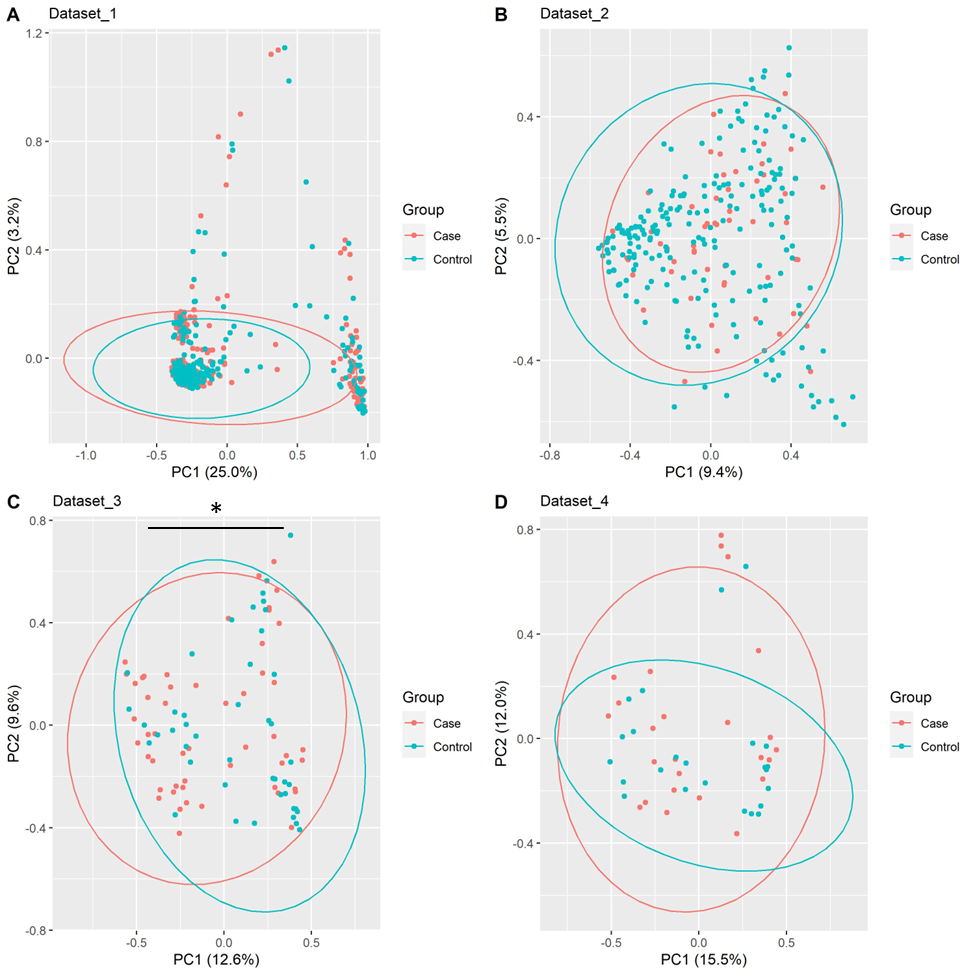


Fig. S16 Beta diversity at genus level in individual datasets. PCoA plots are based on Bray-Curtis dissimilarity which show the differences in the composition of the lung microbiome between cases and controls. Each dot represents the microbiome of a sample. Asterisk indicates a significant result from PERMANOVA. Case: lung tumor tissues, Control: tumor-adjacent normal tissues, *p < 0.05.


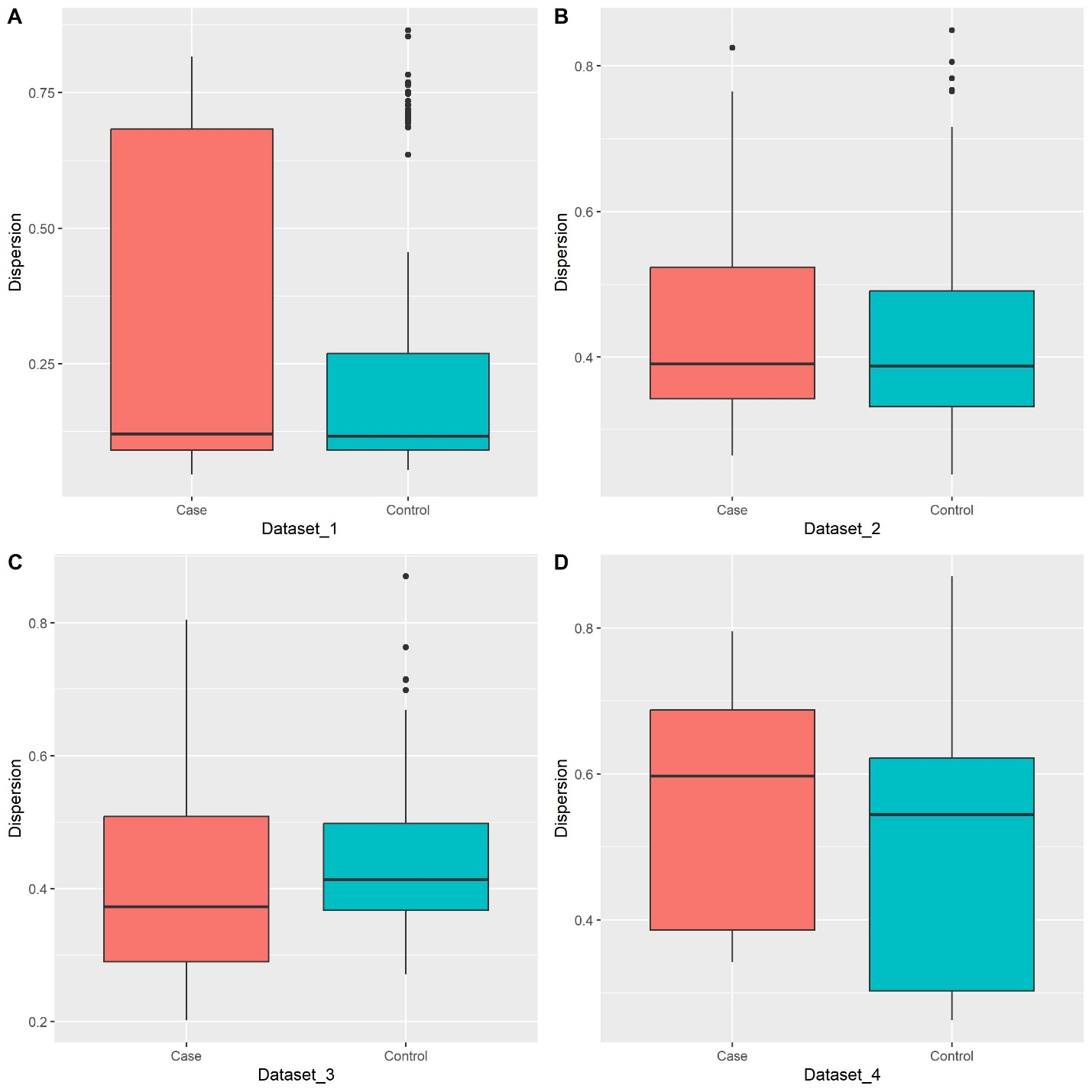


Fig. S17 Beta dispersion at genus level in individual datasets. Boxplots show dispersion within and between groups. The differences between the two groups were tested using ANOVA the results of which were not statistically significant. Case: lung tumor tissues, Control: tumor-adjacent normal tissues.
