# Supplementary figures and images for: Exploring Co-occurrence patterns and microbial diversity in the lung microbiome of patients with non-small cell lung cancer
Source: BMC Microbiol. 2023 Jul 11;23:182. doi: 10.1186/s12866-023-02931-9 (PMC10334658; doi:10.1186/s12866-023-02931-9)

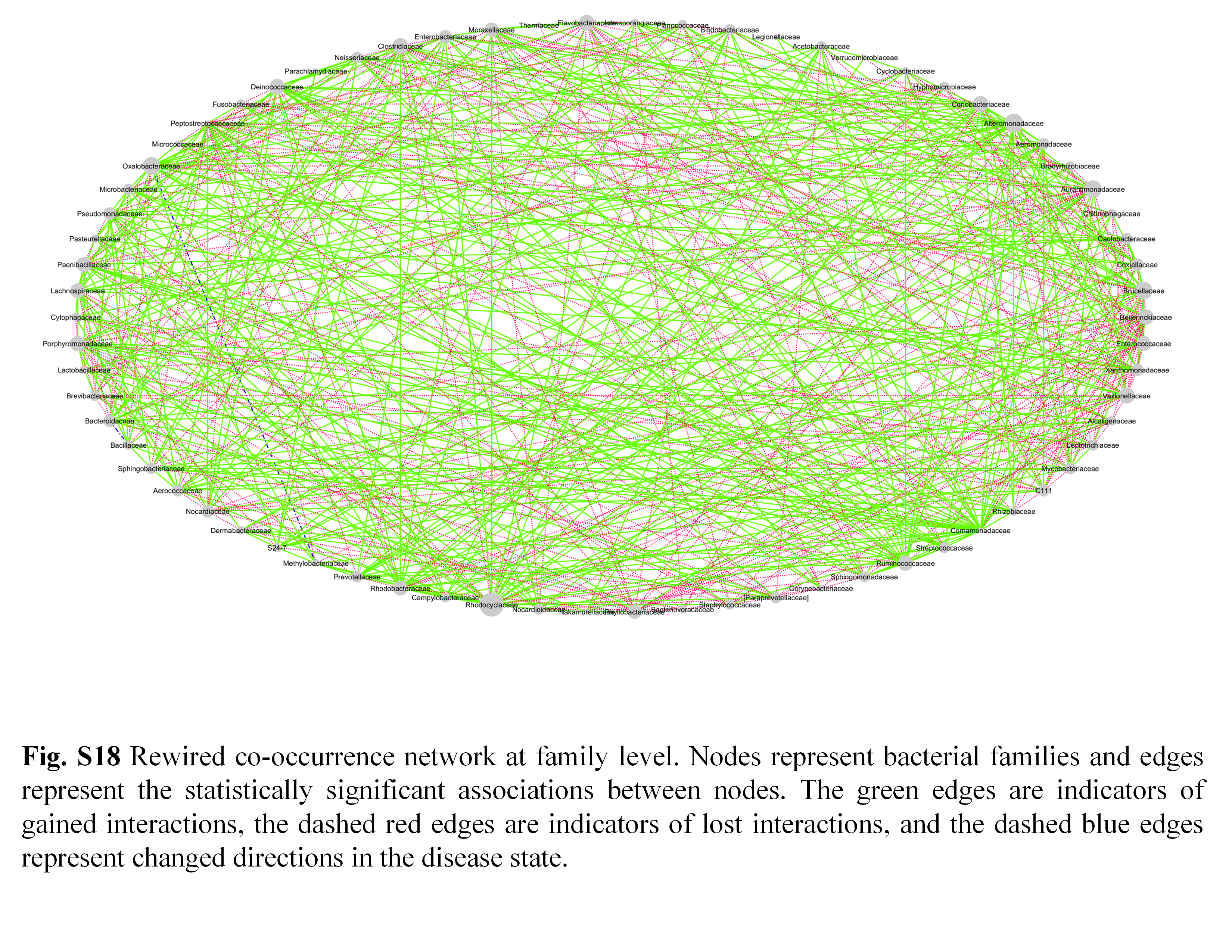

Supplement: Supplementary file 3 — Additional file 3. Rewired co-occurrence network at the family level. [file 12866_2023_2931_MOESM3_ESM.tif]
