# Supplementary material for: Exploring Co-occurrence patterns and microbial diversity in the lung microbiome of patients with non-small cell lung cancer
Source: BMC Microbiol. 2023 Jul 11;23:182. doi: 10.1186/s12866-023-02931-9 (PMC10334658; doi:10.1186/s12866-023-02931-9)
Supplement: Supplementary file 5 — Additional file 5. Values assigned to filtering parameters used in the denoising step in QIIME. [file 12866_2023_2931_MOESM5_ESM.pdf]

Table S3. Values assigned to filtering parameters used in the denoising step in QIIME

	Qiime dada2 denoise-paired				Qiime feature-table filter-samples
	trim-left-f	trim-left-r	trunc-len-f	trunc-len-r	min-frequency
PRJNA472758	25	25	200	180	3000
PRJNA624822	18	18	126	126	-
PRJNA303190	25	25	270	270	3000
PRJNA327258	25	25	270	270	3000
PRJNA647170	17	21	210	210	2000

f: forward read, r: reverse read
